# A Combinatorial Functional Precision Medicine Platform for Rapid Therapeutic Response Prediction in AML

**DOI:** 10.1002/cam4.70401

**Published:** 2024-11-19

**Authors:** Noor Rashidha Binte Meera Sahib, Jameelah Sheik Mohamed, Masturah Bte Mohd Abdul Rashid, Jayalakshmi, Yihao Clement Lin, Yen Lin Chee, Bingwen Eugene Fan, Sanjay De Mel, Melissa Gaik Ming Ooi, Wei‐Ying Jen, Edward Kai‐Hua Chow

**Affiliations:** ^1^ Cancer Science Institute of Singapore National University of Singapore Singapore; ^2^ NUS Centre for Cancer Research (N2CR), Yong Loo Lin School of Medicine National University of Singapore Singapore; ^3^ Department of Pharmacology, Yong Loo Lin School of Medicine National University of Singapore Singapore; ^4^ Department of Haematology‐Oncology, National University Cancer Institute National University Health System Singapore; ^5^ KYAN Technologies Pte Ltd Singapore; ^6^ Department of Haematology Tan Tock Seng Hospital Singapore; ^7^ Lee Kong Chain School of Medicine Nanyang Technological University Singapore; ^8^ Department of Laboratory Medicine Khoo Teck Puat Hospital Singapore; ^9^ Department of Leukemia The University of Texas MD Anderson Cancer Center Houston Texas USA; ^10^ Department of Biomedical Engineering, College of Design and Engineering National University of Singapore Singapore

**Keywords:** AML, combinatorial drug sensitivity, FLT3, functional precision medicine

## Abstract

**Background:**

Despite advances made in targeted biomarker‐based therapy for acute myeloid leukemia (AML) treatment, remission is often short and followed by relapse and acquired resistance. Functional precision medicine (FPM) efforts have been shown to improve therapy selection guidance by incorporating comprehensive biological data to tailor individual treatment. However, effectively managing complex biological data, while also ensuring rapid conversion of actionable insights into clinical utility remains challenging.

**Methods:**

We have evaluated the clinical applicability of quadratic phenotypic optimization platform (QPOP), to predict clinical response to combination therapies in AML and reveal patient‐centric insights into combination therapy sensitivities. In this prospective study, 51 primary samples from newly diagnosed (ND) or refractory/relapsed (R/R) AML patients were evaluated by QPOP following ex vivo drug testing.

**Results:**

Individualized drug sensitivity reports were generated in 55/63 (87.3%) patient samples with a median turnaround time of 5 (4–10) days from sample collection to report generation. To evaluate clinical feasibility, QPOP‐predicted response was compared to clinical treatment outcomes and indicated concordant results with 83.3% sensitivity and 90.9% specificity and an overall 86.2% accuracy. Serial QPOP analysis in a FLT3‐mutant patient sample indicated decreased FLT3 inhibitor (FLT3i) sensitivity, which is concordant with increasing FLT3 allelic burden and drug resistance development. Forkhead box M1 (FOXM1)—AKT signaling was subsequently identified to contribute to resistance to FLT3i.

**Conclusion:**

Overall, this study demonstrates the feasibility of applying QPOP as a functional combinatorial precision medicine platform to predict therapeutic sensitivities in AML and provides the basis for prospective clinical trials evaluating ex vivo‐guided combination therapy.

## Graphical abstract

The Quadratic Phenotypic Optimization Platform (QPOP) allows for the analysis of patient‐specific combinatorial drug sensitivities without prior genetic testing in a rapid manner compared to standard genetic testing. This method can guide clinical decisions and help provided patient care in a timely manner (Created in BioRender. Sahib, N. (2024) BioRender.com/a92n028)

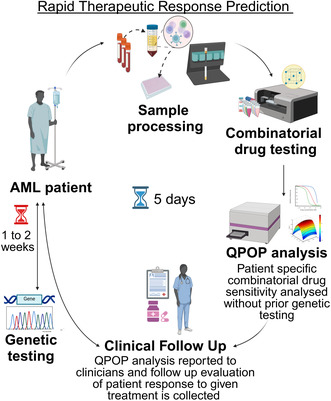

## 1 Introduction

Understanding how various genetic alterations [1, 2, 3] affect acute myeloid leukemia (AML) disease prognosis and influence drug response is paramount to treatment selection, guiding clinicians to adapt therapeutic strategies based on individual genetic profiles and risk factor [4]. Hence, genetic testing for established biomarkers, such as FMS‐like tyrosine kinase 3 (*FLT3*) internal tandem duplication (*FLT3*‐ITD) mutations, are incorporated into standard clinical practice to guide targeted or combinatorial treatment selection [1, 3, 5, 6]. Despite promising results of targeted therapy, the duration of response remains short‐lived due to secondary resistance [7]. The RATIFY trial demonstrated 58.9% complete remission among patients treated with FLT3 targeted inhibitor (FLT3i) midostaurin and chemotherapy. However, half of these patients eventually relapsed [8, 9]. Similarly, gilteritinib monotherapy only showed complete remission in 45%–55% of patients with relapsed and refractory (R/R) *FLT3‐*mutated AML [7]. Treatment effectiveness is limited by the genomic complexity of AML [10] and patients relapse upon gain of new genetic aberrations or from existing co‐mutations that elude treatment [11, 12]. Current risk stratification methods are primarily reliant on cytogenetic findings at initial diagnosis and are based on a standard panel of genetic markers that offer incomplete prognostic information [13]. Induction therapy with targeted molecular treatments based on selected genetic markers continues to have limited long‐term efficacy. The presence of a genetic mutation alone does not necessarily indicate the outcome of treatment [14] due to monotherapy resistance and the heterogeneous nature of AML during disease progression [15]. Discrepancies in predicting clinical outcomes highlight the necessity to refine risk stratification methods [16]. Accurately predicting treatment response in individual patients remains an elusive challenge.

Advances in ex vivo drug sensitivity testing (DST) based on molecular phenotypic responses have facilitated the development of functional precision medicine (FPM) strategies across a range of hematological malignancies [17, 18, 19]. The Extended Analysis for Leukemia/Lymphoma Treatment (EXALT) trial [20] demonstrated the use of imaging‐based single‐cell analysis to assess cell population response upon drug treatment [21]. Similarly, Liebers and team [22], assessed ex vivo responses to various single drugs to determine drug response profiles for individuals [22]. Together with studies [23, 24] interrogating drug‐response prediction methodologies using omics data, these FPM platforms have demonstrated the integration of ex vivo functional test results into a complex tumor board decision [22, 25] process, providing clinical therapy guidance. While these studies show that FPM approaches are beneficial [21], they are usually dependent on single drug sensitivity results that may not recapitulate combinatorial drug sensitivity [26, 27] or may rely on extensive molecular profiling that requires long turnaround time [28, 29, 30]. Hence, there is a need for improved combination treatment selection guidance for patients in a clinically relevant time frame.

The quadratic phenotypic optimization platform (QPOP) is a hybrid experimental–computational precision medicine platform utilizing drug combination sensitivity analysis to determine optimal treatment outcomes [18, 31, 32, 33]. QPOP uses quadratic relationships to correlate the input of predetermined drug candidates and doses to experimentally derived phenotypic outputs of tumor volume reduction or cell viability reduction [34, 35]. Patient drug sensitivity responses to specified test combinations of drugs are fit within an orthogonal array composite design (OACD) [36] model to derive individualized patient‐specific coefficients [37, 38]. These values describe patient‐specific responses to all potential drug combinations, without any prior assumption of mechanism. QPOP has previously been evaluated for its clinical feasibility as an FPM platform for R/R non‐Hodgkin lymphoma (NHL) patients [18] and used to identify drug combinations in bortezomib‐resistant multiple myeloma (MM) cell line models [32]. In this study, we aim to evaluate the clinical applicability of QPOP to identify effective drug combinations for individual AML patients by evaluating the concordance between clinical response against QPOP‐predicted response of the given treatment. We demonstrate QPOP's ability to predict drug sensitivity according to FLT3 mutation status, without prior genomic profiling.

## 2 Methods

### 2.1 Study Design and Patient Cohort

The prospective cohort study aimed to evaluate the ability of applying QPOP as a clinical decision‐making support system in AML patients. This study was conducted between November 26, 2019 to September 15, 2023 and patients were recruited from the National University Hospital of Singapore and Tan Tock Seng Hospital, Singapore. Following institutional review board approvals (IRB: H‐18‐032 and 2019/00271) and informed consent, patients aged 21 years and above diagnosed with AML as defined by the World Health Organization 2017 were enrolled [39]. All subjects gave their informed consent for inclusion before they participated in the study. Peripheral blood (PB) and bone marrow aspirate (BMA) samples were obtained for QPOP analysis. Subjects who were aged below 21 years, pregnant, or HIV positive were excluded.

### 2.2 Patient Sample Preparation and Culture Conditions

Mononuclear cells were isolated from samples using Ficoll‐Paque plus (GE HealthCare) density gradient centrifugation method. Additional blast cell isolation [40, 41] (CD34 or CD33 Microbeads, human, Miltenyi Biotec) was included. For tissue‐banked samples, a dead cell removal kit (Miltenyi Biotec) was used to remove nonviable cells. The samples were plated in 384‐well microtiter plates at a density of 100,000 cells/ml and incubated overnight at 37°C with 5% CO_2_ before drug treatment. Drug combinations were dispensed using an automated liquid dispenser (D300e Digital dispenser, Tecan), and cell viability was measured after 48 h using CellTiter‐Glo Luminescent Cell Viability Assay (Promega) according to the manufacturer's protocol. To ensure samples integrity is retained and to remain viable during treatment duration, primary cells were cultured short‐term in Roswell Park Memorial Institute (RPMI)‐1640 medium supplemented with 10% fetal bovine serum, and 1% penicillin/streptomycin at 5% CO_2_ at 37°C. Flow cytometry analysis at 48 h posttreatment of negative control indicates cell viability and the profile remains similar to the sample origin (Figure S1). Samples were normalized to individual untreated negative controls during data collection as baseline viability scores.

### 2.3 QPOP Analysis and Clinical Application

Ex vivo drug treatment test points were adapted from the OACD by Xu, Jaynes, Ding [37]. Twelve drugs were screened at three different concentrations with a total of 155 test combinations evaluated per run as previously described [18]. Ex vivo drug treatment data were subsequently analyzed using the Optim.AI software platform (KYAN Technologies) to determine top‐ranked combinations with the greatest cell‐killing capability as suggested by control normalized QPOP output values from a ranked list of 531,441 (3^12^) possible drug combinations. Patient‐specific QPOP reports summarizing the top‐ranking combinations were provided to the treating clinicians. Quality assessment of the QPOP assay was evaluated by *Z*‐factor (*Z*′) and strictly standardized mean difference scores. Treatment plan selection was at clinician discretion, independent of QPOP results. Patient risk factor and clinical response outcomes were categorized according to European LeukemiaNet (ELN) criteria 2022 [42]. Clinical response (inclusive of CR, CRi, and PR) or no clinical response (NR) was evaluated post‐induction. The primary endpoint was the feasibility of applying QPOP in the clinical setting for AML as determined by concordance between clinical outcome and QPOP‐predicted response.

### 2.4 RNA Sequencing of Cell Lines

MV4‐11 parental line and MV4‐11‐R (FLT3i‐resistant line) were seeded at 100,000 cells/ml density in 10 cm plates and were treated with midostaurin (FLT3i) and MK2206 (AKT inhibitor) as single drug or in combination. Cell pellets of treated samples were collected 48 h posttreatment. RNA extraction was conducted according to the manufacturer's protocol (QIAGEN RNeasy kit). RNA sequencing of biological triplicates was performed on the Illumina NovaSeq platform (NovogeneAIT Genomics, Singapore). Gene set enrichment analysis (GSEA) Kyoto Encyclopedia of Genes and Genomes (KEGG) analysis was used to identify differentially expressed genes and pathways that contribute to drug resistance (Venn diagram: https://csbg.cnb.csic.es/BioinfoGP/venny.html [43]).

### 2.5 Western Blot Assay

Whole‐cell lysates were extracted from cell pellets with a RIPA lysis buffer with Pierce protease and phosphatase inhibitor cocktail tablets. The lysates were resolved on 8% or 10% polyacrylamide gels and transferred onto a polyvinylidene fluoride (PVDF) membrane before being probed with primary and secondary antibodies. The chemiluminescence signal was visualized on the ChemiDoc Imaging System, and the relative band intensities were quantified with the Bio‐Rad Image Lab software. The primary antibodies used are described in Table S1. β‐actin was used as a loading control.

### 2.6 Statistical Analysis

Statistical analyses were conducted on the GraphPad Prism v8.0.2. Comparisons within groups were performed using ordinary one‐way ANOVA and the respective pairwise comparison as recommended by the software at 5% significance level. Statistical analyses of the QPOP regression model were performed on Optim.AI software together with the establishment of the model. The adjusted R^2^ value was used to determine QPOP‐generated model robustness. All data shown are means and standard deviations of three biological replicates unless otherwise stated. Statistical tests evaluated for QPOP concordance indicate a 95% confidence interval (CI) and Fisher's exact test was used to determine significance.

## 3 Results

### 3.1 Patient Demographics and Technical Feasibility of QPOP in AML

**FIGURE 1:**
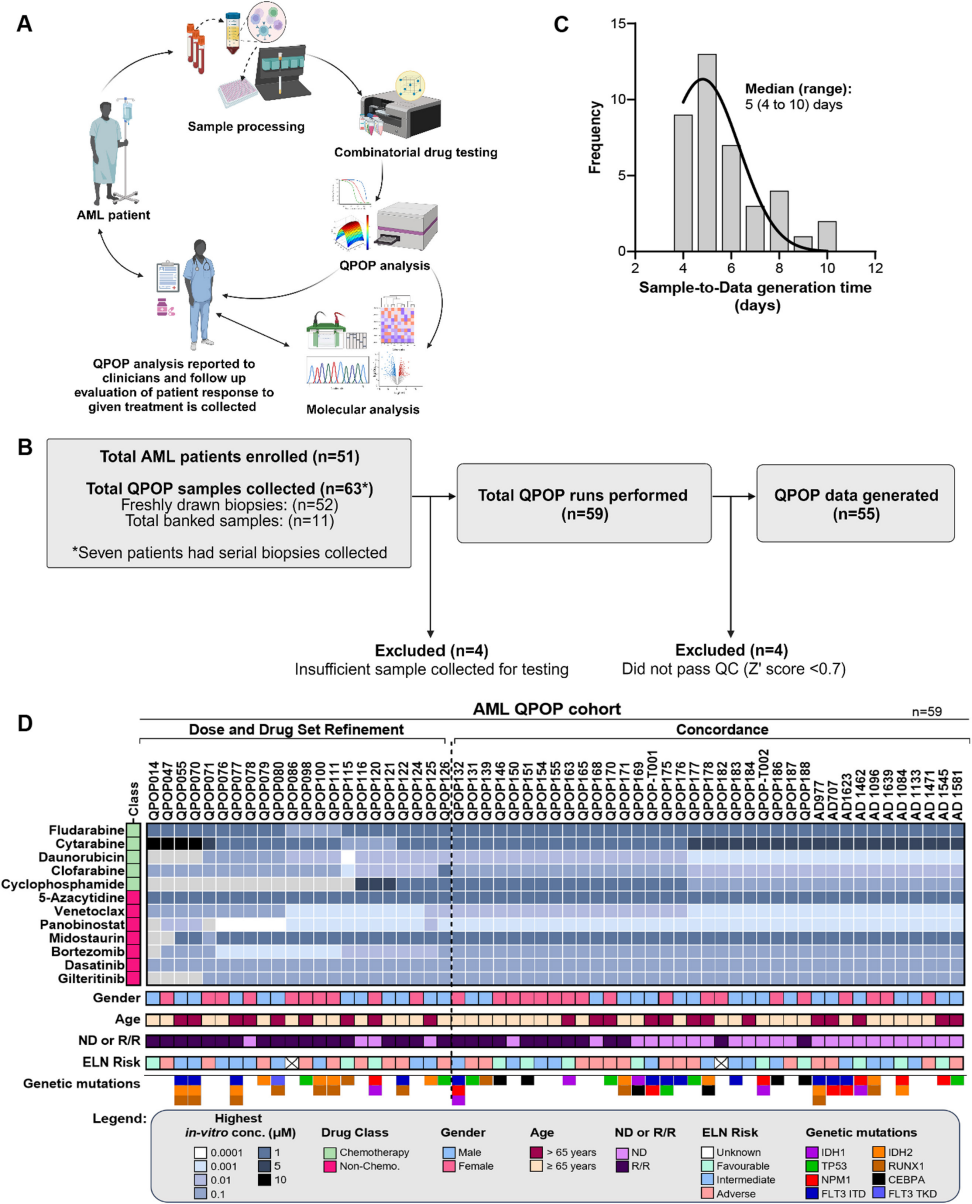
Overall workflow of QPOP study and summary of patient characteristics. (A) The schematic figure indicates the workflow from patient sample collection to QPOP analysis and clinical feedback (Created in BioRender. Meera Sahib, N. (2024) https://BioRender.com/a92n028). (B) Fifty‐one unique AML patients were recruited for QPOP drug combination testing and 55 out of 59 QPOP runs had data generated. (C) Median turnover time for QPOP report was 5 days which is suitable in a clinically relevant timeframe. (D) The heatmap summarizes patient characteristics, and drug panel and concentrations used for each individual QPOP run. The “highest *in‐vitro* conc.” represents the range in which the highest drug concentration used for each drug per QPOP run falls within.

Fifty‐one patients were enrolled in this study of whom 28 were newly diagnosed (ND) and 23 were R/R patients (at first QPOP run), with a median age of 60 years (range 20–82 years old). Seven patients had biopsies collected at multiple time points for QPOP analysis. Sixty‐three AML samples were collected including 52 samples of freshly drawn biopsies for prospective QPOP testing and 11 banked tissue samples collected for retrospective QPOP analysis. Four AML samples were excluded due to insufficient sample volume at collection. Fifty‐nine QPOP runs were performed (33 BMA and 26 PB). Twenty‐eight QPOP runs were conducted at point of diagnosis and 31 at relapse. QPOP analysis generated evaluable data in 55 runs with four samples excluded due to poor *Z*′ score values (*Z*′ value < 0.7). The overall study workflow is as indicated in Figure 1A,B. Seventeen samples were excluded from the final QPOP concordance evaluation due to the following conditions: patients lost to follow‐up (*n* = 4), patients who passed away prior to any treatment post‐QPOP sample collection (*n* = 8), and patients prescribed treatment outside of the evaluable QPOP drug panel (*n* = 5). Thirty‐eight of 55 cases (69.1%) were evaluated for clinical concordance. The overall median turnaround time for QPOP results from time of sample collection to QPOP data generation was 5 days (range: 4–10 days; Figure 1C).

An AML‐specific 12‐drug panel was optimized for the cohort according to clinician recommendations, comprising clinically approved chemotherapy and targeted therapy options. Drug choices (Table S2) and dosing concentrations were adjusted based on physician feedback. Drug concentrations were selected based on either IC_5_–IC_10_ or 5%–10% of maximum serum concentration (C_max_). 10% C_max_ was identified from published studies [44] and was used as the maximal cutoff concentration for experiments as it is believed to be the concentration level achievable at the target tissue [38, 45]. Hence, for QPOP analysis, the highest concentrations were maintained to be 10% of C_max_ or at IC_10_ to ensure clinical relevance of dosing levels and to prevent overrepresentation of the drugs in QPOP analysis (Table S3). Based on prior studies [18], optimization of the drug dosing utilized in the ex vivo DST improves clinical accuracy of QPOP analysis. Thus, the cumulative single drug sensitivity of the first 22 samples was used to adjust QPOP drug dosing (Figure S2). Twenty‐nine of the 38 evaluable QPOP runs were further analyzed for their concordance after optimizing for dosing concentrations. The summary of patient demographic and drugs used is shown in Figure 1D.

### 3.2 Clinical Concordance of QPOP Analysis to Treatment Outcomes

We aimed to assess the QPOP's ability to predict patient drug sensitivity. Based on receiver operating characteristic (ROC) curve analysis, a 0.65 normalized cell viability (NCV) score was determined as the cutoff point for QPOP‐predicted evaluation [46]. This threshold reflects an optimal balance between sensitivity and specificity for predicting drug sensitivity accurately. NCV score below 0.65 indicates a significant reduction in cell survival in response to the drug combination, correlating with clinical sensitivity, whereas scores above 0.65 suggest resistance. This cutoff enhances QPOP prediction accuracy while minimizing false positives, aligning with clinical response patterns. Hence, patient NCV scores, of a given treatment above 0.65 were considered predicted non‐responders, and below 0.65 was considered predicted responders.

**FIGURE 2:**
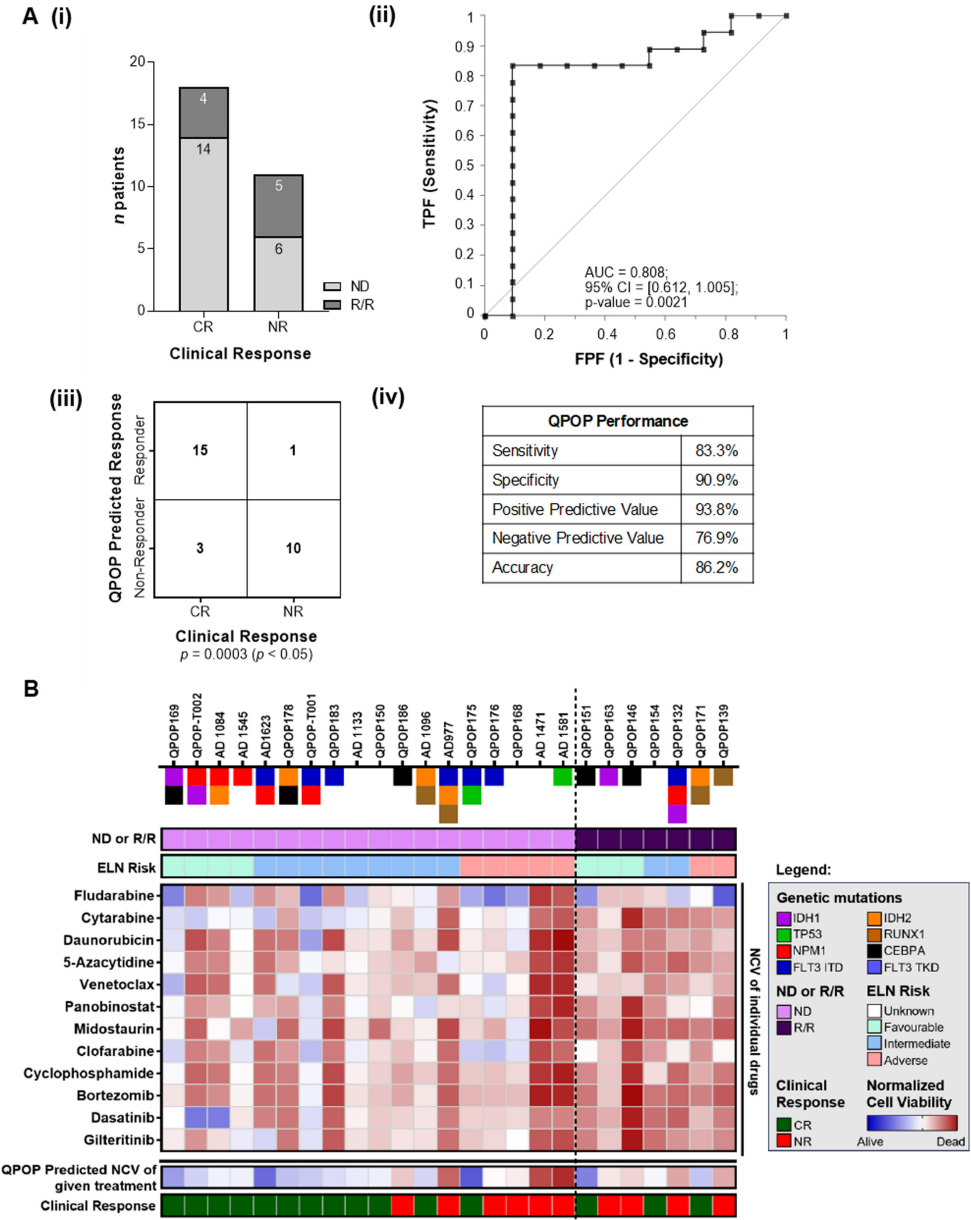
Clinical concordance of 29 evaluable patients with standard QPOP drug set and single drug viability of concordant patients. (A) (i) Overall clinical response for the 29 QPOP runs with evaluable data and standardized QPOP dosing and drug set. (ii) The ROC curve (receiver operating characteristic curve) indicates the performance of QPOP has an AUC value of 0.808. (iii) and (iv) indicate the overall QPOP‐predicted response with respect to clinical outcomes and the statistical evaluation of the response. (B) Average normalized cell viability (NCV) scores of single drugs of the 25 patients with concordant clinical data were analyzed and patients with adverse risk or R/R had an overall poorer NCV score compared to ND or those with favorable risk. QPOP‐predicted NCV scores of combination therapy given and actual clinical response was concordant. NCV of individual drugs does not fully reflect combinatorial response.

Of 29 evaluable QPOP runs, 20 were ND patients and 9 were R/R. Eighteen of these patients (62.1%) had a CR and 11 patients (37.9%) had NR. Fourteen out of 20 (70.0%) ND patients responded to the given treatment and 4 out of 9 (44.4%) R/R responded to the given treatment. The predictive value of QPOP score (AUC = 0.808, CI: 0.612–1.005) was significant (*p* = 0.0021). QPOP accurately predicted 15 out of 18 responders and 10 out of 11 non‐responders with a sensitivity of 83.3% (CI: 57.7%–95.5%), specificity of 90.9% (CI: 57.2%–99.5%), positive predictive value (PPV) of 93.75% (CI: 67.8%–99.7%), and negative predictive value (NPV) of 76.9% (CI: 46.0%–93.8%) (Figure 2A). Comparison of clinical concordance of QPOP following dosage refinement compared to the total QPOP clinical cohort data set (Figure S3A) reaffirms the importance of accounting for cumulative single drug sensitivity prior to utilization of QPOP as a clinical decision support platform. Evaluating all single drug NCV of patients with concordant results indicated R/R patients had a poorer viability scores overall than ND patients. Similarly, patients with adverse ELN risk were more likely to have poorer response to each drug as shown in Figure 2B. However, single drug sensitivity alone did not reflect drug combination effects and their outcomes [47].

### 3.3 Frequently Occurring Combinations Compared to Azacytidine and Venetoclax

**FIGURE 3:**
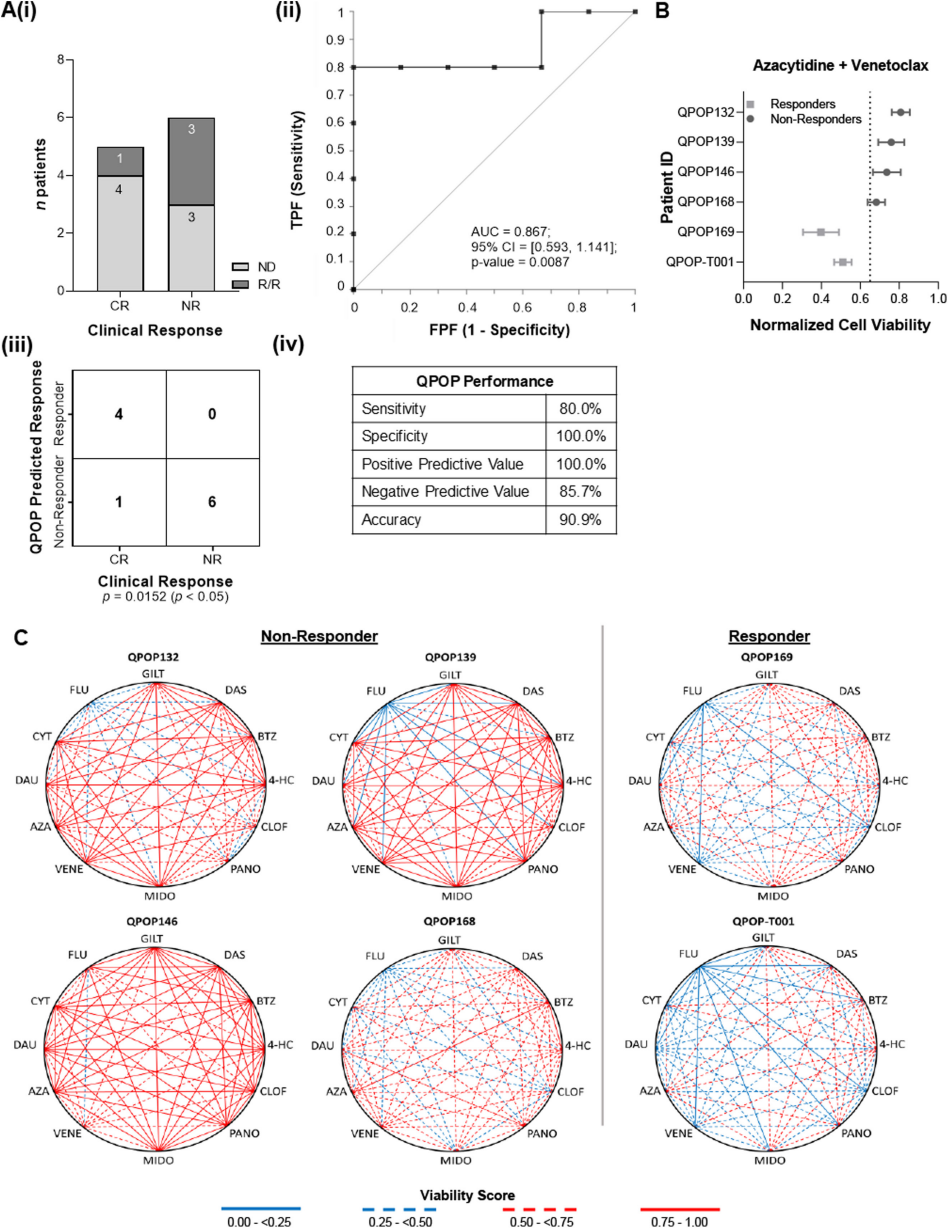
Clinical concordance of AZA‐VEN‐treated patients. (A) (i) Overall clinical response of 11 patients treated with azacytidine and venetoclax drug combination post QPOP. (ii) The ROC curve (receiver operating characteristic curve) indicates the performance of QPOP has an AUC value of 0.867. (iii) and (iv) indicate the QPOP‐predicted response with respect to clinical outcomes to AZA–VEN treatment and the statistical evaluation of the response. (B) The Forrest plot indicates the normalized cell viability of six patients treated with AZA–VEN and patients with lower cell viability were predicted to be clinical responders. Clinical outcomes are concordant and reflective of QPOP‐predicted outcomes. (C) Polygonograms of six representative patients indicate all two drug interactions specific to each individual patient. All four clinical non‐responders had poor viability scores (> 0.5) compared to two clinical responders to AZA–VEN who had better viability scores (< 0.5).

The doublet combination of azacytidine‐venetoclax (AZA‐VEN) is widely prescribed for both ND and R/R patients ineligible for intensive chemotherapy [48, 49]. Of the 29 patients evaluated, AZA‐VEN treatment was the most administered (*n* = 11, 37.9%). Further evaluating QPOP's potential as a clinical decision support tool, the clinical concordance of QPOP to AZA‐VEN treatment was analyzed. Five (45.5%) patients had CR, whereas 6 (54.5%) patients had NR. Among the seven ND patients treated with AZA‐VEN, four patients responded (57.1%) while only one in four R/R patients responded to AZA‐VEN (25%). QPOP analysis was able to accurately identify four of the responders and six of the non‐responders with a sensitivity of 80.0% (CI: 28.4%–99.5%) and specificity of 100% (CI: 54.1%–100%) (*p* = 0.0152). The predictive value of QPOP score (AUC = 0.867, CI: 0.593–1.141) was also significant (*p* = 0.0087). The PPV of QPOP was 100% (CI: 39.8%–100%) and the NPV of QPOP was 85.7% (CI: 51.0%–97.2%) (Figure 3A). Polygonograms used to visualize QPOP‐predicted interactions between all two‐drug combinations indicated lower predicted NCVs observed among AZA‐VEN sensitive patients who were clinical responders. This suggests that QPOP was able to stratify non‐responders and responders for patients treated with AZA‐VEN (Figure 3B,C).

**FIGURE 4:**
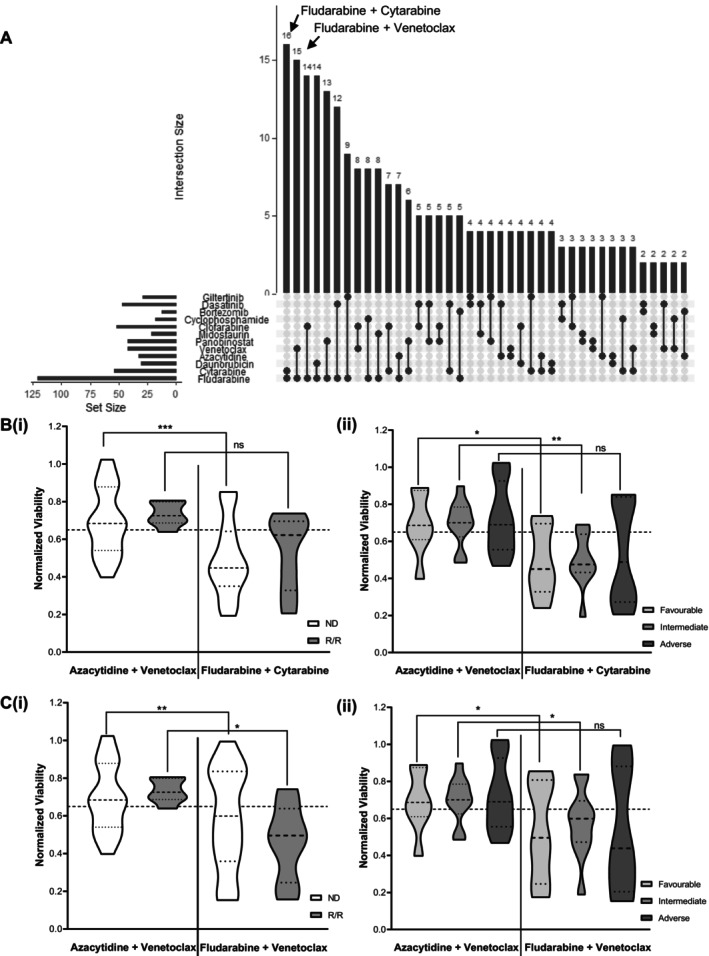
Fludarabine‐based combinations among most frequently occurring top‐ranked combinations. (A) Upset plot indicating the most frequently occurring top‐ranking two‐drug combinations among 25 evaluable patient data identified as fludarabine and cytarabine followed by fludarabine and venetoclax. (B) Comparison of AZA–VEN standard of care to top ranking fludarabine and cytarabine combination based on (i) diagnosis stage (ND: *p* = 0.0003, R/R: *p* = 0.0590 with paired *t*‐test) and (ii) ELN risk (favorable risk: *p* = 0.0205, intermediate risk: *p* = 0.0051, adverse risk: *p* = 0.0662 with paired *t*‐test). (C) Comparison of AZA–VEN standard of care to top ranking fludarabine and venetoclax combination based on (i) diagnosis stage (ND: *p* = 0.0087, R/R: *p* = 0.0189 with paired t‐test) and (ii) ELN risk (favorable risk: *p* = 0.0352, intermediate risk: *p* = 0.0333, adverse risk: *p* = 0.0748 with paired *t*‐test).

In addition to predicting response for individuals, QPOP may be useful in identifying novel combination therapies effective in the wider AML population. We evaluated all top 10 ranking two‐drug combinations in the 25 patients and identified fludarabine and cytarabine as the most frequently occurring doublet combination suggested for patients followed by fludarabine and venetoclax (Figure 4A). Fludarabine is often used in combination with high‐dose cytarabine and granulocyte colony‐stimulating factor (FLAG) for adverse risk patients [50]. FLAG is commonly used for intensive induction management in combination with idarubicin or more recently with venetoclax and has been associated with high CR rate [50, 51, 52, 53]. Identifying appropriate treatment strategies is important for adverse cytogenetic and R/R patients where standard treatments may be less effective [54]. AZA‐VEN treatment has been indicated to be less effective in R/R patients and those with adverse risk cytogenetics compared to ND patients with favorable ELN risk [55, 56]. Hence, we compared the QPOP scores of fludarabine–cytarabine and fludarabine–venetoclax combinations with respect to AZA‐VEN. Fludarabine–cytarabine combination was predicted to be significantly more sensitive among ND patients compared to AZA‐VEN. Additionally, this combination was predicted to be more effective in patients with intermediate and favorable ELN risk than those with adverse risk (Figure 4B). Fludarabine–venetoclax was predicted to be more sensitive than AZA‐VEN regardless of ND or R/R status and was expected to be more sensitive in patients with favorable risk and intermediate risk (Figure 4C). While the *p* value for fludarabine‐based treatment in the adverse risk group compared to AZA‐VEN‐treated adverse risk group was not significant (*p* = 0.0748), patients were generally more sensitive to the fludarabine‐based combinations and a larger cohort may evaluate the advantage of fludarabine‐based combinations in adverse risk patients. This suggests QPOP may be used to suggest alternate treatment combinations beneficial for R/R patients with limited options and prevent the use of unnecessary treatments that may not be effective.

### 3.4 QPOP Prediction of FLT3 Inhibitor Sensitivity in AML

**FIGURE 5:**
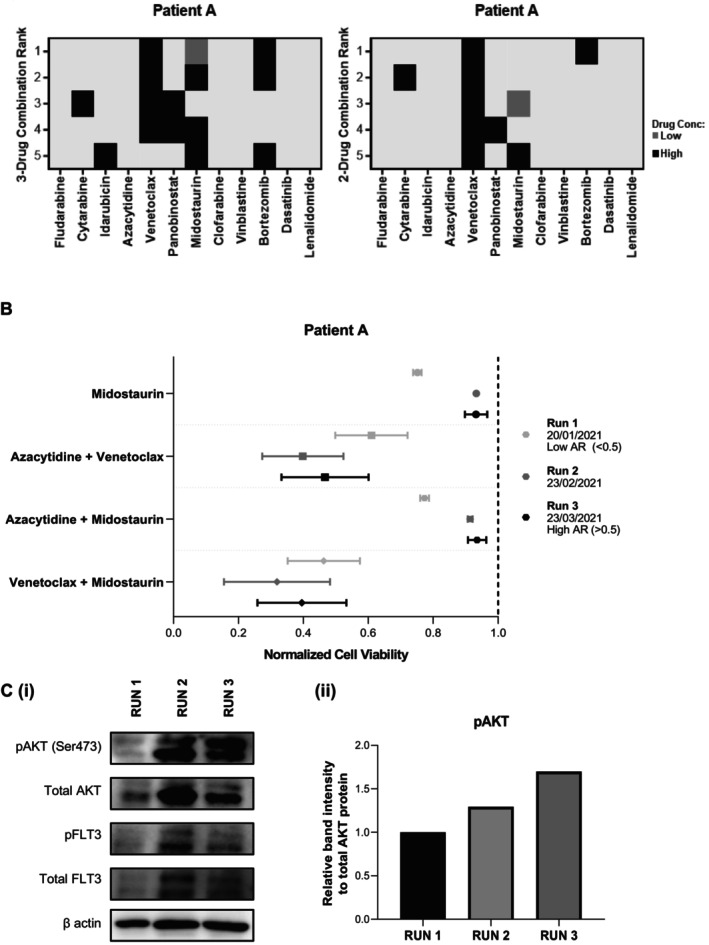
Targeted therapy identification with QPOP in a case study of Patient A. (A) QPOP accurately identified midostaurin‐based combinations among top‐ranking two‐ and three‐drug combinations in patient A who displayed FLT3‐ITD mutations in clinical testing. (B) Consecutive QPOP runs on patient A over 3 months indicated higher cell viability scores to FLT3i midostaurin and its combinations which was concordant with patient's increasing resistance to therapy and increasing FLT3‐ITD mutation allelic ratio. (C) Increasing resistance to FLT3i‐based combinations was associated with an increase in phosphorylated AKT expression in patient A as seen in western blot analysis and quantification.

Response to biomarker‐linked targeted treatment can be variable [57]. Interestingly, without prior genomic information, QPOP was able to identify that a patient (patient A) was responsive to FLT3i, with midostaurin‐based drug combinations appearing as top‐ranking drug combinations suitable for the patient (Figure 5A). Subsequent genetic testing of patient A's sample confirmed the acquisition of FLT3‐ITD mutation with a low allelic ratio upon relapse at the time of the initial QPOP run. Similar concordance of FLT3 mutational status and QPOP‐predicted FLT3i sensitivity was observed in other patients (Figure S4). QPOP was able to identify FLT3i sensitivity among patients without FLT3 mutations as well (Figure S4E). FLT3i has been known to show efficacy even among patients without an FLT3‐ITD/TKD mutation [58]. This further reiterates QPOP's ability to predict effective drug response without reliance on genomic biomarkers.

Given the potential of QPOP to predict FLT3i sensitivity or resistance, we further evaluated eight AML cell lines with varying FLT3 mutational status (Figure S5). As expected, QPOP‐predicted greater sensitivity to midostaurin or gilteritinib among FLT3‐ITD‐mutant cell lines and not among FLT3 WTcell lines or in FLT3i‐resistant cell line MV4‐11‐R. Comparing parental cell line MV4‐11 and its isogenic FLT3i‐resistant cell line MV4‐11‐R, QPOP was able to distinguish the varied drug sensitivity. This further confirms that QPOP‐predicted outputs can match genomic‐based drug sensitivities while identifying potential drug resistances that cannot be determined from standard biomarker testing.

Drug combination strategies including FLT3 targeted therapy have been investigated for deeper and more durable responses, with the azacitidine and midostaurin combination having been investigated in phase I/II trials [59]. The FLT3‐ITD mutation is associated with the constitutive activation of FLT3 resulting in activation of downstream targets involved in various signaling pathways [60] that may be associated with drug resistance [61]. Downstream signaling pathways like phosphatidylinositol‐3‐kinase/protein kinase B (PI3K/AKT) as well as RAS–ERK pathways have been linked with FLT3 pathway signaling. Hence, downstream signaling mediators of the FLT3 pathway were interrogated in OCI‐AML2, MV4‐11, and MV4‐11‐R following treatment with azacytidine or midostaurin individually and in combination. These cell lines were chosen to evaluate the effect of FLT3i on these pathways in the context of changes in FLT3 mutation status and acquired drug resistance. While pAKT expression was present among all cell lines, upon midostaurin treatment both OCI‐AML2 and MV4‐11 showed a downregulation of pAKT expression. However, we observed persistent pAKT expression in resistant cell line MV4‐11‐R upon midostaurin treatment both as a monotherapy and in combination with azacytidine (Figure S5D).

Concurrently, serial QPOP runs across 3 months for patient A indicated diminishing sensitivity to FLT3i midostaurin upon each consecutive run. This increase in resistance to FLT3i was concordant with the patient developing a higher *FLT3* allelic burden as per clinical testing (Figure 5B). Hence, given the observed increase in pAKT in resistant cell lines, we evaluated pAKT expression in patient A who had serial QPOP runs and increasing FLT3i resistance. In our study, we observed that patient A had increased phosphorylated AKT (pAKT) expression (Figure 5C) with increasing FLT3 mutational burden and the development of resistance to FLT3i overtime. The observed similarities to increased pAKT found in the serial primary patient samples lend weight to the potential role of AKT signaling in resistance to FLT3i. These findings also suggest that QPOP can potentially streamline and guide clinical decision‐making through early identification of drug resistance in patients and allows early treatment adjustments to other potential alternative therapies.

### 3.5 Role of pAKT‐FOXM1 Pathway in FLT3 Inhibitor Resistance

**FIGURE 6:**
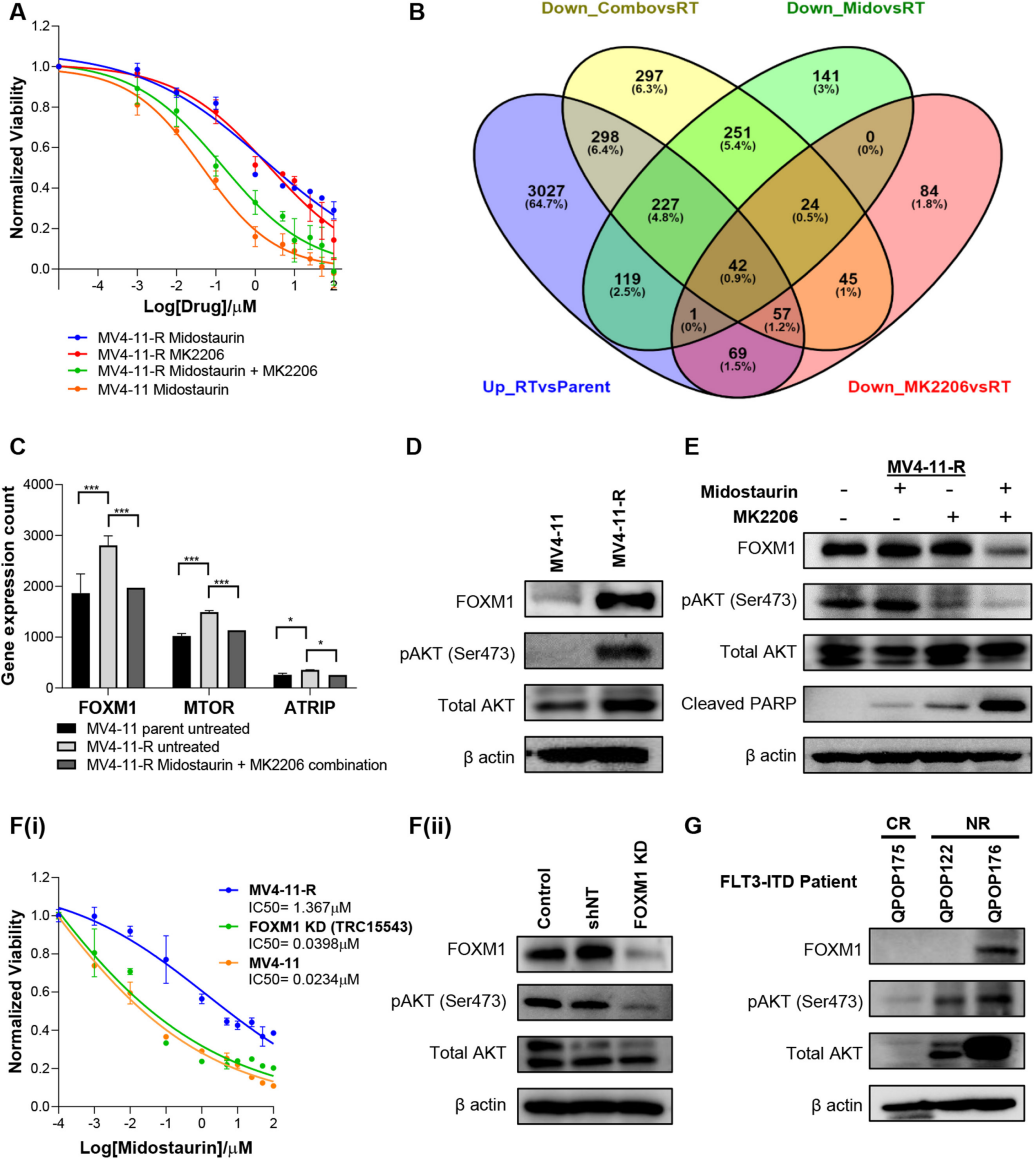
Resistance to FLT3 inhibitors and pAKT‐FOXM1 pathway. (A) IC_50_ curves of midostaurin and AKT inhibitor MK2206 as a single drug and combination in MV4‐11‐R cell line in comparison to MV4‐11 parental line. (B) Venn diagram indicating the common differentially expressed genes among the different treatment groups in MV4‐11‐R and MV4‐11. (C) FOXM1 was identified as the most differentially expressed gene among targets involved in both AKT and DDR pathways. (D) Western blot analysis shows increased FOXM1 protein expression in parent vs isogenic resistant line. (E) Combination of midostaurin and AKT inhibitor MK2206 resulted in decreased FOXM1 and pAKT expression and an increase in cleaved PARP expression. This indicates that AKT inhibitors restore sensitivity to midostaurin treatment in resistant cell line. (F) Loss of function of *FOXM1* gene restores sensitivity to midostaurin. FOXM1 KD line showed (i) reduced IC_50_ concentration of midostaurin compared to control MV4‐11‐R and indicated (ii) reduced pAKT expression compared to control. (G) FLT3 patients with known clinical non‐response to FLT3 inhibitor therapy indicated high pAKT expression. While patients with complete response to FLT3 inhibitors had low pAKT expression.

The molecular mechanisms contributing to the upregulation of AKT pathway in FLT3‐ITD‐mutant AML are still not completely understood especially with regards to resistance to targeted therapy. Given the potential role of AKT in FLT3i resistance observed in both cell lines and patient samples, the mechanism by which resistance occurred was further explored. Sensitivity to midostaurin in MV4‐11‐R cells was analyzed in the presence or absence of MK2206, a chemical inhibitor of AKT. Inhibition of AKT by MK2206 restored midostaurin sensitivity in MV4‐11‐R cells (Figure 6A) indicating the potential role of AKT in mediating FLT3i resistance. RNA‐sequencing analysis of MV4‐11, control MV4‐11‐R, MV4‐11‐R cells treated with midostaurin only, and AKT‐inhibited MV4‐11‐R with and without midostaurin was performed to interrogate the mechanisms by which AKT‐mediate FLT3i resistance. To understand how the AKT pathway restores sensitivity to midostaurin, gene expression of key downstream targets of AKT pathway in resistant lines were compared to the parental line. Genes that were significantly upregulated upon gain of resistance and downregulated upon loss of AKT expression with MK2206 and midostaurin in MV4‐11‐R (not monotherapy) were evaluated based on differential gene expression analysis (Figure 6B). Additionally, KEGG pathway analysis [62] on AKT‐inhibited MV4‐11‐R treated with midostaurin revealed significant downregulation of various pathways that are involved in the cellular DNA damage response (DDR) (Figure S6). The downregulation of genes in these pathways can impair cellular response to DNA damage and significantly increase the sensitivity of cancer cells to chemotherapy. Among the differentially expressed genes, downstream targets of both AKT and DDR pathways were evaluated (Figure S7). Among these genes, Forkhead box protein M1 (FOXM1) was observed to be the most differentially expressed and downregulated upon treatment with AKT inhibitor and midostaurin (Figure 6C). AKT pathway regulation of DDR is well studied [63, 64] and FOXM1 has been indicated to regulate the transcription of multiple DDR proteins and is crucial in cancer development and chemoresistance [63, 64, 65, 66, 67].

FOXM1 expression has been associated with PI3K‐AKT pathway and the upregulation of AKT or FOXM1 individually has been noted to promote chemoresistance in FLT3‐ITD AML [64, 67]. Current studies suggest that FOXM1 may reciprocally promote AKT activation in a positive feedback loop to cause chemoresistance [63]. However, the link between FLT3i resistance and FOXM1‐AKT feedback loop has not been experimentally confirmed in relation to FLT3‐ITD AML. Hence, we evaluated the link between FLT3i resistance and FOXM1‐AKT. Western blot analysis of MV4‐11 and MV4‐11‐R in the presence of midostaurin and MK2206 revealed an increase in protein expression of both pAKT and FOXM1 in resistant line compared to the parental MV4‐11 (Figure 6D) and downregulation of pAKT and FOXM1 upon treatment of midostaurin and MK2206 in MV4‐11‐R (Figure 6E). These results reiterate RNA‐sequencing data that FOXM1 may contribute to AKT‐mediated midostaurin resistance. We further evaluated the role of FOXM1 in midostaurin resistance through *in‐vitro* loss‐of‐function studies following FOXM1 gene knockdown (FOXM1‐KD) in MV4‐11‐R cell line. FOXM1‐KD resulted in restored sensitivity to midostaurin in MV4‐11‐R cell line with IC_50_ reduced from 1.367 μM to 0.0398 μM, comparable to midostaurin IC_50_ in MV4‐11 (0.0234 μM) which was associated with decreased pAKT expression (Figure 6F). To determine if increased co‐expression of FOXM1 and pAKT can also be observed in clinical non‐responders to FLT3i treatment, western blot analysis was conducted in three primary patient samples with known FLT3 mutations and clinical response. Patients who had poor clinical outcomes following FLT3i treatment (QPOP122 and QPOP176) had increased pAKT expression compared to complete responder (QPOP175). In addition to increased pAKT, QPOP176 also had increased FOXM1 expression compared to the other two patients (Figure 6G). Thus, while FOXM1 may contribute to FLT3i resistance in the clinical setting, there may be other molecular mechanisms that drive AKT signaling and subsequent FLT3i resistance.

## 4 Discussion

Sequencing efforts have been extensively improved, allowing the identification of a wide spectrum of mutations in AML. These efforts have been useful in guiding clinical decision‐making as the presence of specific mutations can indicate increased sensitivity to specific standard therapy regimens [1]. For example, venetoclax sensitivity in patients maybe specified by patient genetic mutations. ASXL1 [68] or NPM1 mutations have been linked to venetoclax treatment sensitivity [69]. NPM1 patients, however, presenting with certain co‐mutations like FLT3‐ITD, RAS, TP53, or WT1 can be resistant to venetoclax treatment [69]. While genetic profiling can guide decisions on sensitivity of certain drugs, with more treatment options increasingly developed and more genomic data available through the standard of care testing as disease progresses, clinicians face a challenge in determining suitable treatments for patients [70], especially for patients with complex genetic or cytogenetics. This is especially more complicated for R/R patients who may lose or gain mutations through disease progression and maybe not respond to certain therapies. Hence, ex vivo drug sensitivity testing efforts can help overcome this challenge of identifying appropriate treatment options for R/R patients whose genomic biomarkers alone may no longer be reflective of their treatment response. Additionally, there is an increasing need for combinatorial treatment approaches to effectively treat the disease by targeting genetically diverse subclones, survival signals from the tumor microenvironment, and intrinsic feedback mechanisms within the tumor, all of which contribute to disease relapse [71].

Patient treatment selection tailored to individual needs can eliminate exposure to multiple rounds of chemotherapeutics [72, 73, 74]. Various FPM strategies that combine molecular/phenotypic profiling data and DST have been developed to refine drug response predictions, fostering improved treatment efficacy and risk stratification [20, 21, 22, 23, 75, 76, 77, 78]. These platforms have allowed a greater understanding of existing molecular biomarkers and drug response enabling the repurposing of established drugs [79] and guiding clinicians in identifying personalized treatment selection including those with limited options available [80]. FPM studies utilizing flow cytometry such as BH3 profiling [81, 82] and imaging‐based strategies [83, 84, 85] provide single‐cell resolution of cell‐type specific responses that distinguish between malignant and nonmalignant cell responses accounting for potential toxicity^30^. These studies indicate the feasibility of ex vivo DST platforms in the clinical setting to guide treatment selection for AML patients and the potential of FPM to standard clinical decision support in AML [76]. However, current FPM methods are limited in terms of combination drug sensitivity predictions as they are commonly based on single drug sensitivity scores or utilize pre‐designed existing combinations [4, 19, 24, 71]. Furthermore, some of these platforms rely on existing large population datasets and extensive genomic profiling to build predictive value [3, 18, 72, 83, 86]. The need for specialized computational tools to analyze large data sets may be resource and time‐intensive, limiting scalability, and accessibility [87].

FPM platforms that evaluate drug response based on phenotypic changes rather than single molecular targets may provide more expansive treatment recommendations [88]. Variability in clinical response rates [89, 90] necessitate improved treatment selection and identification of responders [51]. Here we demonstrate the ability of QPOP to recommend combinatorial drug treatments for patients including targeted therapy regardless of individual drug sensitivity or existing biomarkers but instead, treatment recommendations are specific to patient combinatorial drug sensitivity profiles. Moreover, QPOP not only provides patient‐specific responses in a clinically relevant timeframe [31, 37] but has also indicated strong clinical response concordance. QPOP maybe a valuable tool in guiding clinical decisions [91, 92] to accurately identify responders, preventing unnecessary treatment, and may even provide more effective alternatives like fludarabine and cytarabine that may be more effective in R/R AML or high‐risk AML. Despite overall clinical concordance being reliably evaluated, unlike the previous clinical study of QPOP in NHL, no patients were guided by QPOP to receive treatment. A phase II trial comparing physician‐guided therapy response to QPOP‐guided treatment response is necessary to determine the clinical benefit of this platform for R/R AML patients. Furthermore, the current study did not include immunotherapy drugs nor consider the effects of normal immune cells on drug response. QPOP can be applied toward any quantifiable phenotypic readout, including apoptosis and xenograft tumor volume reduction, as the basis for combinatorial drug response evaluation [32]. As such, we are currently optimizing a flow cytometry immunophenotyping‐based application of QPOP [93] that can evaluate response to immunotherapy and highlights the blast‐specific cell viability readout in relation to the complete immune environment without having to isolate the blast cell population.

Elucidation of molecular mechanisms of AML pathogenesis has led to the emergence of targeted agents for AML treatment [94]. While the presence of a targeted biomarker may not reflect drug sensitivity, understanding targets involved in resistance signaling pathways allows us to identify potentially novel therapeutic targets. FLT3‐ITD mutations have been associated with multiple signaling pathways, yet how these pathways contribute to drug resistance is still not clear [95]. In this study, we corroborated previous findings that resistance to FLT3i arises from the downstream targets of FLT3 such as the PI3K‐AKT pathway [60, 96]. Here we additionally find that the development of FLT3i resistance is associated with an upregulation of pAKT expression that maybe correlated to FOXM1 expression. The AKT/FOXM1 axis may contribute to midostaurin resistance by promoting cell survival and proliferation despite FLT3 inhibition. The AKT/FOXM1 regulation loop has been shown to contribute to venetoclax resistance in AML [63]. Inactivation of either AKT or FOXM1 signaling was shown to disrupt this loop. Similarly, we show that loss of function of either AKT or FOXM1 restored sensitivity to FLT3i. The upregulation of genes associated with the DDR pathway has been known to result in the evasion of detection by the repair pathway and checkpoints inducing chemotherapy resistance [97]. Utilizing AKT or FOXM1 inhibitors in combination with FLT3i [98] may shift the balance of therapy‐induced DDR from survival toward apoptosis by inhibiting key regulators of the DDR and may overcome resistance [95, 99]. In this study, we highlight the role of the FLT3, AKT, and FOXM1 regulation loop in drug resistance and the need for combinatorial drug therapy to overcome resistance using combinatorial drug therapy such as AKT or FOXM1 inhibitors with FLT3 inhibitors. This further emphasizes the need to use functional precision medicine platforms like QPOP to determine suitable drug combinations for patients in a mechanism‐agnostic manner.

QPOP is beneficial for use as a clinical decision support tool with 86.2% accuracy in predicting response to treatments in AML in a clinically relevant timeframe. Our study offers a rationale to evaluate QPOP‐guided treatment approach in clinical settings examining both new and established drug combinations for AML, including more targeted therapy options. Finally, this work provides further support for the utilization of FPM platforms in AML as clinical decision‐support tools to help improve treatment outcomes.

## Author Contributions

**Noor Rashidha Binte Meera Sahib:** conceptualization (equal), data curation (lead), formal analysis (lead), investigation (lead), methodology (lead), project administration (lead), validation (lead), visualization (lead), writing – original draft (lead), writing – review and editing (lead). **Jameelah Sheik Mohamed:** data curation (equal). **Masturah Bte Mohd Abdul Rashid:** formal analysis (supporting), software (equal). data curation (equal), project administration (equal). **Yihao Clement Lin:** data curation (equal), project administration (equal). **Yen Lin Chee:** investigation (equal), project administration (equal), resources (equal). **Bingwen Eugene Fan:** investigation (equal), project administration (equal), resources (equal). **Sanjay De Mel:** conceptualization (equal), writing – review and editing (supporting). **Melissa Gaik Ming Ooi:** conceptualization (equal), investigation (equal), project administration (equal), resources (equal), writing – review and editing (supporting). **Wei‐Ying Jen:** conceptualization (equal), investigation (equal), project administration (equal), resources (equal), writing – review and editing (supporting). **Edward Kai‐Hua Chow:** conceptualization (lead), funding acquisition (lead), investigation (equal), project administration (equal), resources (equal), software (lead), supervision (lead), writing – original draft (equal), writing – review and editing (lead).

## Ethics Statement

All subjects gave their informed consent as per the National Health Group (NHG) DSRB institutional review board approvals (IRB: H‐18‐032 and 2019/00271) for inclusion before they participated in the study.

## Conflicts of Interest

M.O. received honoraria from Jenssen, Novartis, Astellas, AbbVie, Pfizer Amgen, and Bristol‐Myers Squibb. E.K.H.C. is a shareholder of KYAN Technologies.

## Supporting information


Data S1.

## Data Availability

All data associated with this study are in the paper or the Supporting Information.
