# Supplementary material for: A Combinatorial Functional Precision Medicine Platform for Rapid Therapeutic Response Prediction in AML
Source: Cancer Med. 2024 Nov 19;13(22):e70401. doi: 10.1002/cam4.70401 (PMC11574777; doi:10.1002/cam4.70401)
Supplement: Supplementary file 1 — Data S1. [file CAM4-13-e70401-s001.pptx]

## Slide 1
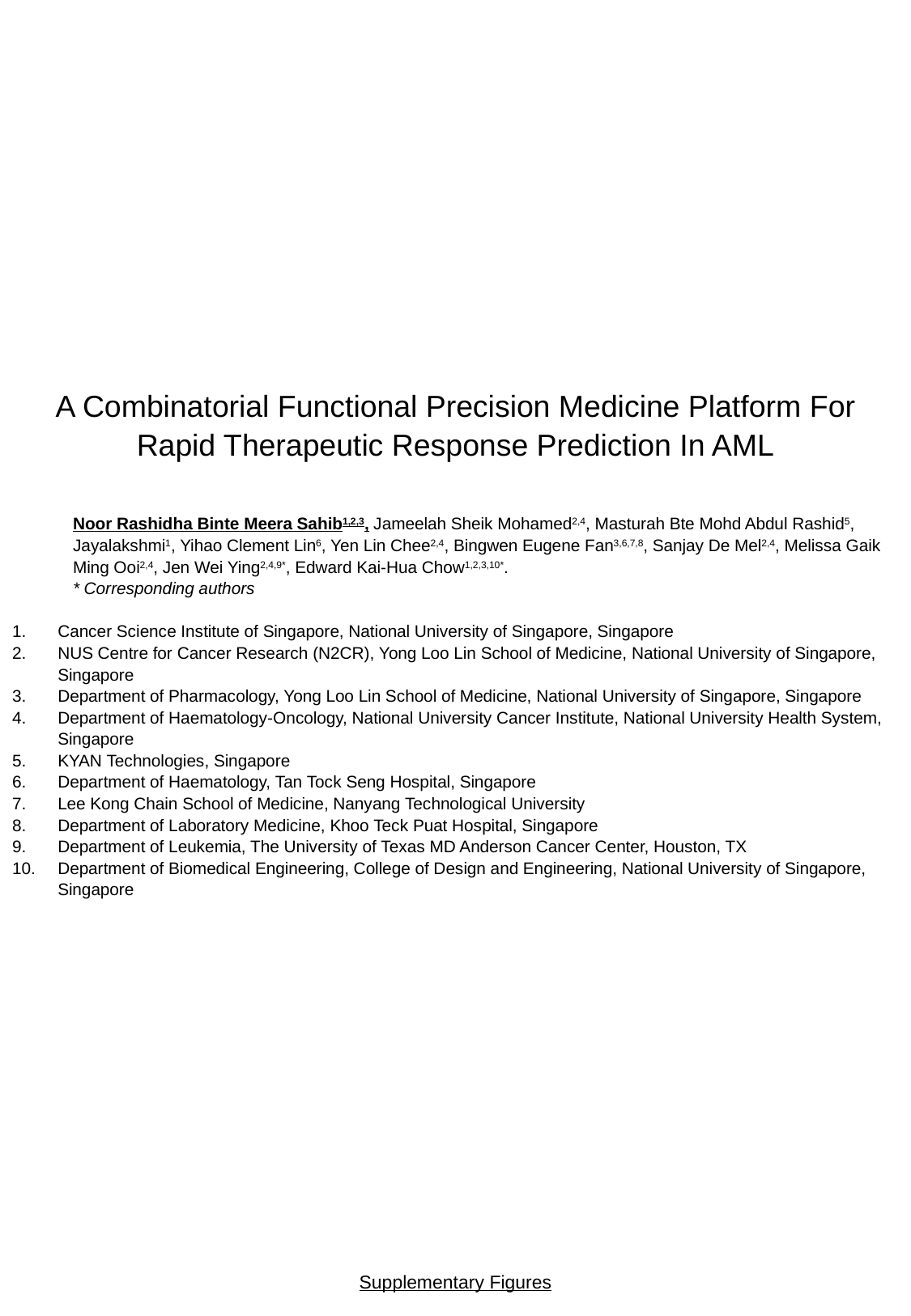

A Combinatorial Functional Precision Medicine Platform For Rapid Therapeutic Response Prediction In AML
Noor Rashidha Binte Meera Sahib1,2,3, Jameelah Sheik Mohamed2,4, Masturah Bte Mohd Abdul Rashid5, Jayalakshmi1, Yihao Clement Lin6, Yen Lin Chee2,4, Bingwen Eugene Fan3,6,7,8, Sanjay De Mel2,4, Melissa Gaik Ming Ooi2,4, Jen Wei Ying2,4,9*, Edward Kai-Hua Chow1,2,3,10*.
* Corresponding authors
Cancer Science Institute of Singapore, National University of Singapore, Singapore
NUS Centre for Cancer Research (N2CR), Yong Loo Lin School of Medicine, National University of Singapore, Singapore
Department of Pharmacology, Yong Loo Lin School of Medicine, National University of Singapore, Singapore
Department of Haematology-Oncology, National University Cancer Institute, National University Health System, Singapore
KYAN Technologies, Singapore
Department of Haematology, Tan Tock Seng Hospital, Singapore
Lee Kong Chain School of Medicine, Nanyang Technological University
Department of Laboratory Medicine, Khoo Teck Puat Hospital, Singapore
Department of Leukemia, The University of Texas MD Anderson Cancer Center, Houston, TX
Department of Biomedical Engineering, College of Design and Engineering, National University of Singapore, Singapore
Supplementary Figures

## Slide 2
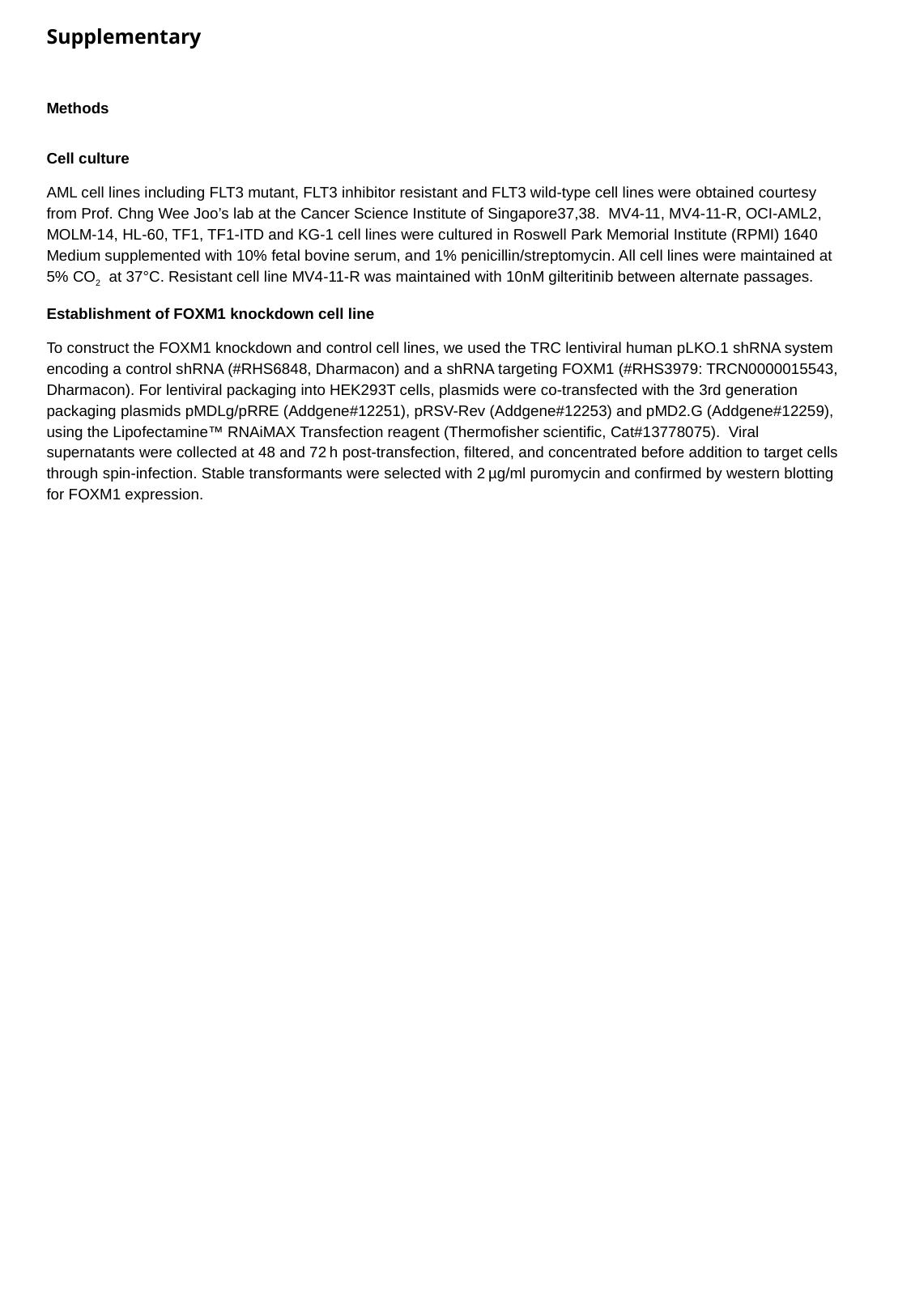

Supplementary
Methods
Cell culture
AML cell lines including FLT3 mutant, FLT3 inhibitor resistant and FLT3 wild-type cell lines were obtained courtesy from Prof. Chng Wee Joo’s lab at the Cancer Science Institute of Singapore37,38. MV4-11, MV4-11-R, OCI-AML2, MOLM-14, HL-60, TF1, TF1-ITD and KG-1 cell lines were cultured in Roswell Park Memorial Institute (RPMI) 1640 Medium supplemented with 10% fetal bovine serum, and 1% penicillin/streptomycin. All cell lines were maintained at 5% CO2 at 37°C. Resistant cell line MV4-11-R was maintained with 10nM gilteritinib between alternate passages.
Establishment of FOXM1 knockdown cell line
To construct the FOXM1 knockdown and control cell lines, we used the TRC lentiviral human pLKO.1 shRNA system encoding a control shRNA (#RHS6848, Dharmacon) and a shRNA targeting FOXM1 (#RHS3979: TRCN0000015543, Dharmacon). For lentiviral packaging into HEK293T cells, plasmids were co-transfected with the 3rd generation packaging plasmids pMDLg/pRRE (Addgene#12251), pRSV-Rev (Addgene#12253) and pMD2.G (Addgene#12259), using the Lipofectamine™ RNAiMAX Transfection reagent (Thermofisher scientific, Cat#13778075). Viral supernatants were collected at 48 and 72 h post-transfection, filtered, and concentrated before addition to target cells through spin-infection. Stable transformants were selected with 2 µg/ml puromycin and confirmed by western blotting for FOXM1 expression.

## Slide 3
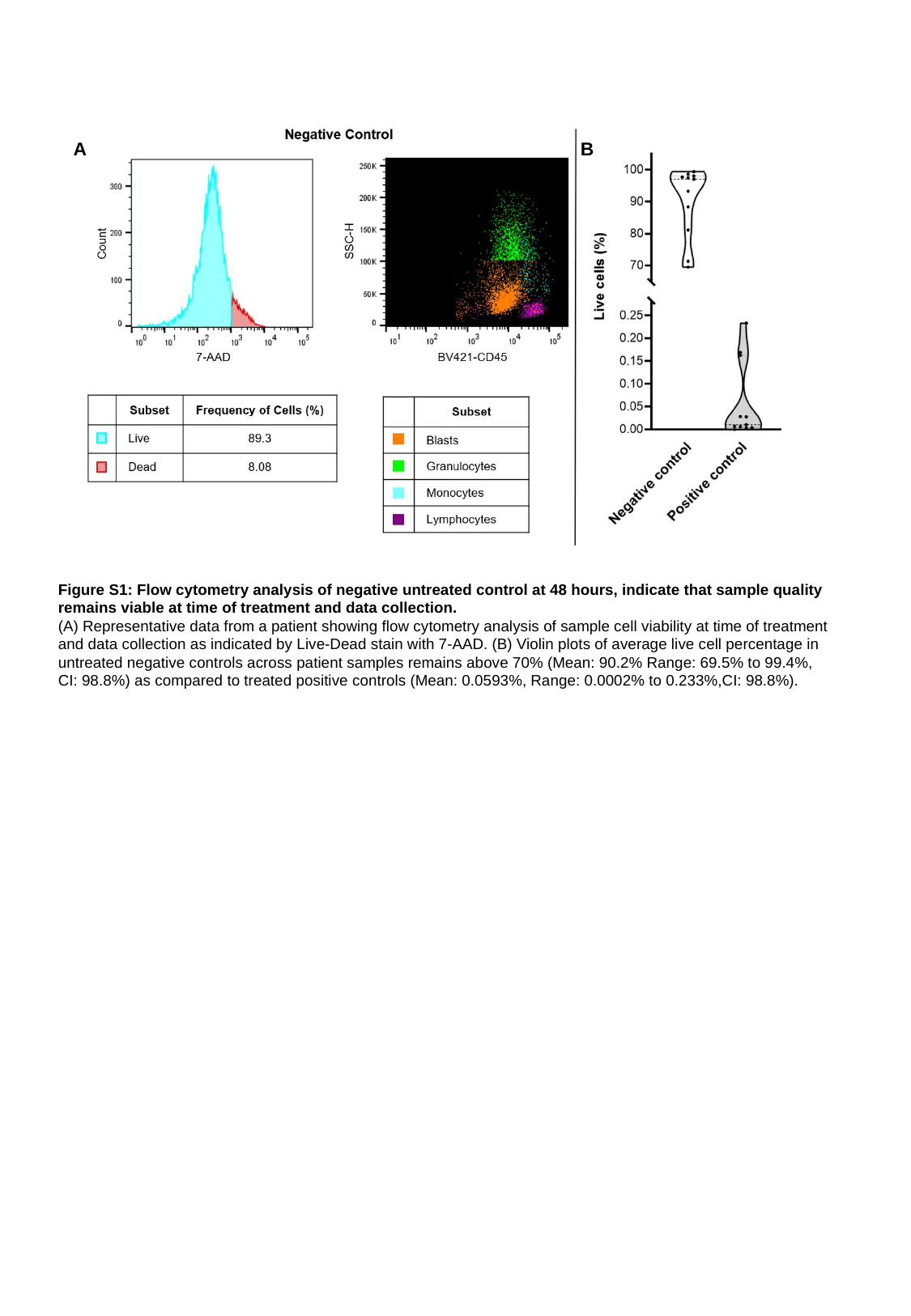

B
A
Figure S1: Flow cytometry analysis of negative untreated control at 48 hours, indicate that sample quality remains viable at time of treatment and data collection.
(A) Representative data from a patient showing flow cytometry analysis of sample cell viability at time of treatment and data collection as indicated by Live-Dead stain with 7-AAD. (B) Violin plots of average live cell percentage in untreated negative controls across patient samples remains above 70% (Mean: 90.2% Range: 69.5% to 99.4%, CI: 98.8%) as compared to treated positive controls (Mean: 0.0593%, Range: 0.0002% to 0.233%,CI: 98.8%).

## Slide 4
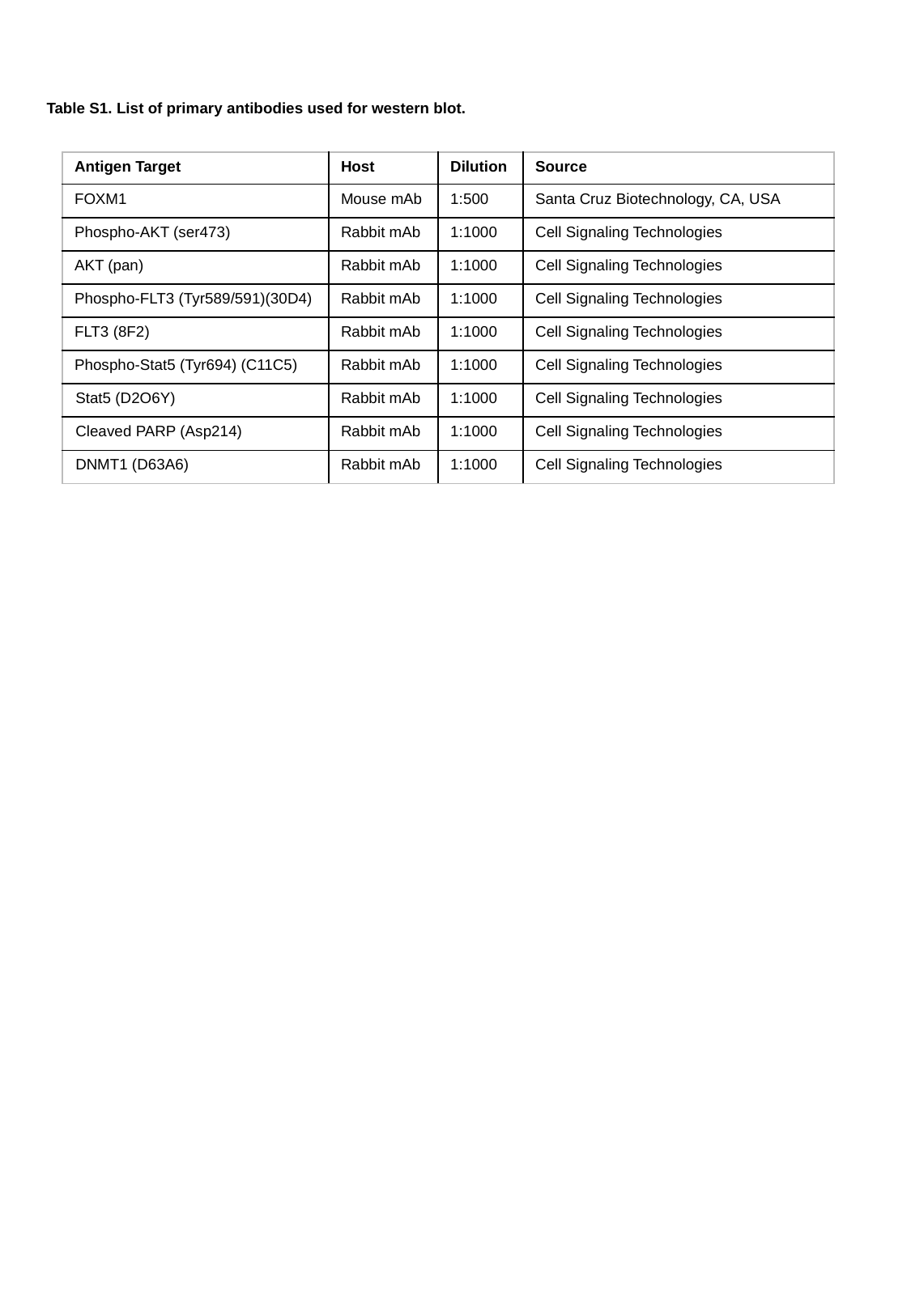

Table S1. List of primary antibodies used for western blot.
| Antigen Target | Host | Dilution | Source |
| --- | --- | --- | --- |
| FOXM1 | Mouse mAb | 1:500 | Santa Cruz Biotechnology, CA, USA |
| Phospho-AKT (ser473) | Rabbit mAb | 1:1000 | Cell Signaling Technologies |
| AKT (pan) | Rabbit mAb | 1:1000 | Cell Signaling Technologies |
| Phospho-FLT3 (Tyr589/591)(30D4) | Rabbit mAb | 1:1000 | Cell Signaling Technologies |
| FLT3 (8F2) | Rabbit mAb | 1:1000 | Cell Signaling Technologies |
| Phospho-Stat5 (Tyr694) (C11C5) | Rabbit mAb | 1:1000 | Cell Signaling Technologies |
| Stat5 (D2O6Y) | Rabbit mAb | 1:1000 | Cell Signaling Technologies |
| Cleaved PARP (Asp214) | Rabbit mAb | 1:1000 | Cell Signaling Technologies |
| DNMT1 (D63A6) | Rabbit mAb | 1:1000 | Cell Signaling Technologies |

## Slide 5
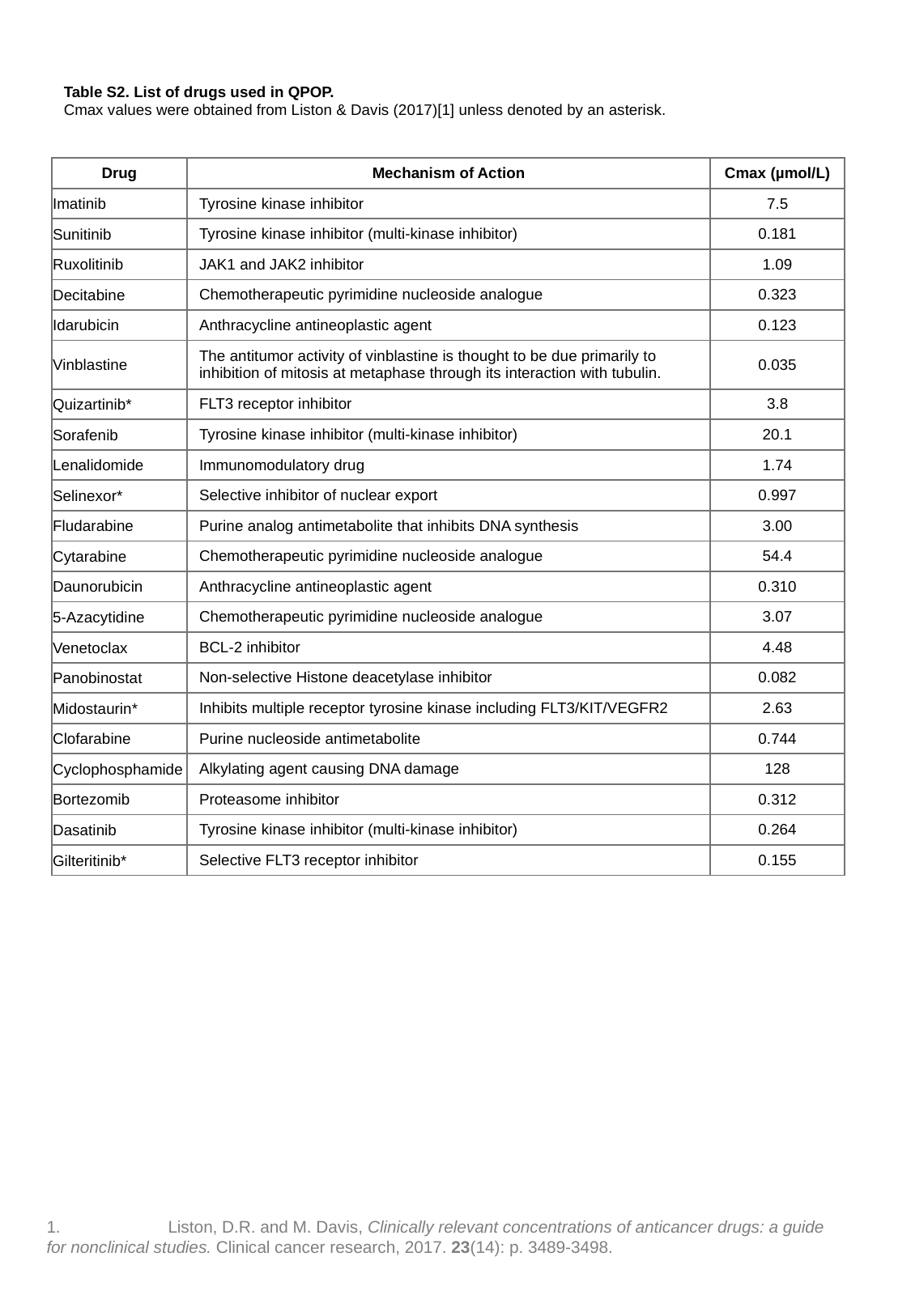

Table S2. List of drugs used in QPOP.
Cmax values were obtained from Liston & Davis (2017)[1] unless denoted by an asterisk.
| Drug | Mechanism of Action | Cmax (µmol/L) |
| --- | --- | --- |
| Imatinib | Tyrosine kinase inhibitor | 7.5 |
| Sunitinib | Tyrosine kinase inhibitor (multi-kinase inhibitor) | 0.181 |
| Ruxolitinib | JAK1 and JAK2 inhibitor | 1.09 |
| Decitabine | Chemotherapeutic pyrimidine nucleoside analogue | 0.323 |
| Idarubicin | Anthracycline antineoplastic agent | 0.123 |
| Vinblastine | The antitumor activity of vinblastine is thought to be due primarily to inhibition of mitosis at metaphase through its interaction with tubulin. | 0.035 |
| Quizartinib\* | FLT3 receptor inhibitor | 3.8 |
| Sorafenib | Tyrosine kinase inhibitor (multi-kinase inhibitor) | 20.1 |
| Lenalidomide | Immunomodulatory drug | 1.74 |
| Selinexor\* | Selective inhibitor of nuclear export | 0.997 |
| Fludarabine | Purine analog antimetabolite that inhibits DNA synthesis | 3.00 |
| Cytarabine | Chemotherapeutic pyrimidine nucleoside analogue | 54.4 |
| Daunorubicin | Anthracycline antineoplastic agent | 0.310 |
| 5-Azacytidine | Chemotherapeutic pyrimidine nucleoside analogue | 3.07 |
| Venetoclax | BCL-2 inhibitor | 4.48 |
| Panobinostat | Non-selective Histone deacetylase inhibitor | 0.082 |
| Midostaurin\* | Inhibits multiple receptor tyrosine kinase including FLT3/KIT/VEGFR2 | 2.63 |
| Clofarabine | Purine nucleoside antimetabolite | 0.744 |
| Cyclophosphamide | Alkylating agent causing DNA damage | 128 |
| Bortezomib | Proteasome inhibitor | 0.312 |
| Dasatinib | Tyrosine kinase inhibitor (multi-kinase inhibitor) | 0.264 |
| Gilteritinib\* | Selective FLT3 receptor inhibitor | 0.155 |
1.	Liston, D.R. and M. Davis, Clinically relevant concentrations of anticancer drugs: a guide for nonclinical studies. Clinical cancer research, 2017. 23(14): p. 3489-3498.

## Slide 6
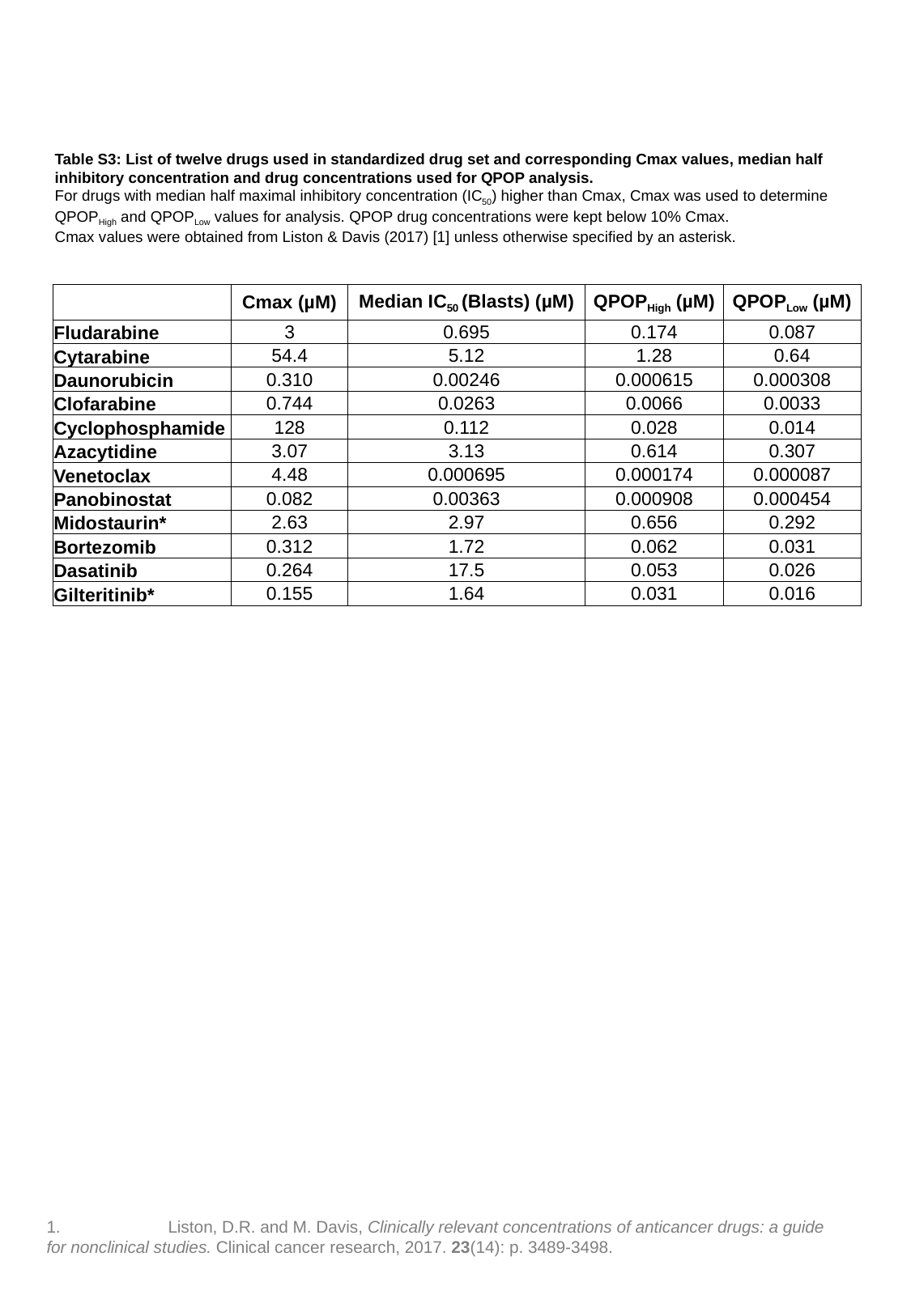

Table S3: List of twelve drugs used in standardized drug set and corresponding Cmax values, median half inhibitory concentration and drug concentrations used for QPOP analysis.
For drugs with median half maximal inhibitory concentration (IC50) higher than Cmax, Cmax was used to determine QPOPHigh and QPOPLow values for analysis. QPOP drug concentrations were kept below 10% Cmax.
Cmax values were obtained from Liston & Davis (2017) [1] unless otherwise specified by an asterisk.
| | Cmax (µM) | Median IC50 (Blasts) (µM) | QPOPHigh (µM) | QPOPLow (µM) | |
| --- | --- | --- | --- | --- | --- |
| Fludarabine | 3 | 0.695 | 0.174 | 0.087 | |
| Cytarabine | 54.4 | 5.12 | 1.28 | 0.64 | |
| Daunorubicin | 0.310 | 0.00246 | 0.000615 | 0.000308 | |
| Clofarabine | 0.744 | 0.0263 | 0.0066 | 0.0033 | |
| Cyclophosphamide | 128 | 0.112 | 0.028 | 0.014 | |
| Azacytidine | 3.07 | 3.13 | 0.614 | 0.307 | |
| Venetoclax | 4.48 | 0.000695 | 0.000174 | 0.000087 | |
| Panobinostat | 0.082 | 0.00363 | 0.000908 | 0.000454 | |
| Midostaurin\* | 2.63 | 2.97 | 0.656 | 0.292 | |
| Bortezomib | 0.312 | 1.72 | 0.062 | 0.031 | |
| Dasatinib | 0.264 | 17.5 | 0.053 | 0.026 | |
| Gilteritinib\* | 0.155 | 1.64 | 0.031 | 0.016 | |
1.	Liston, D.R. and M. Davis, Clinically relevant concentrations of anticancer drugs: a guide for nonclinical studies. Clinical cancer research, 2017. 23(14): p. 3489-3498.

## Slide 7
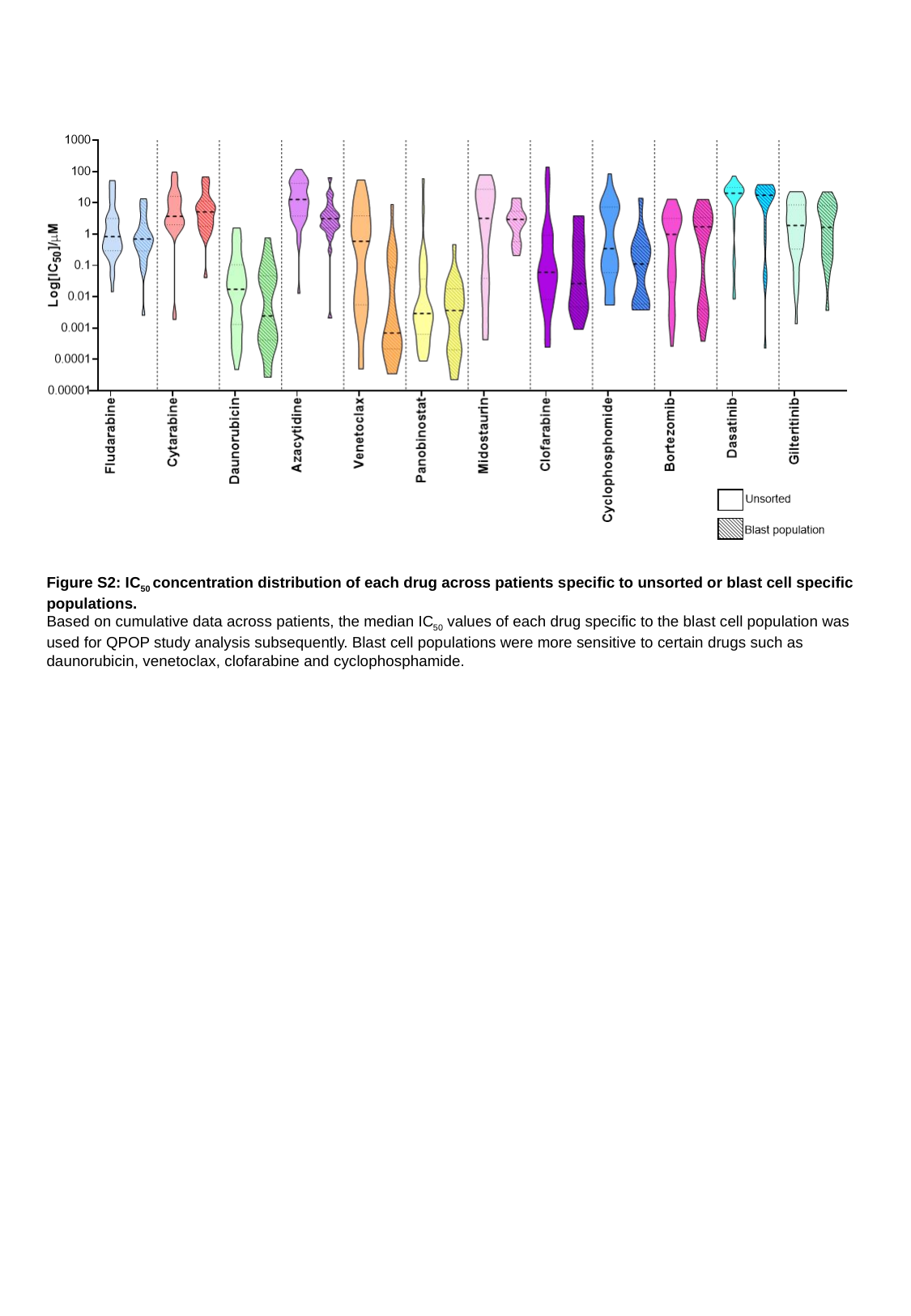

Figure S2: IC50 concentration distribution of each drug across patients specific to unsorted or blast cell specific populations.
Based on cumulative data across patients, the median IC50 values of each drug specific to the blast cell population was used for QPOP study analysis subsequently. Blast cell populations were more sensitive to certain drugs such as daunorubicin, venetoclax, clofarabine and cyclophosphamide.

## Slide 8
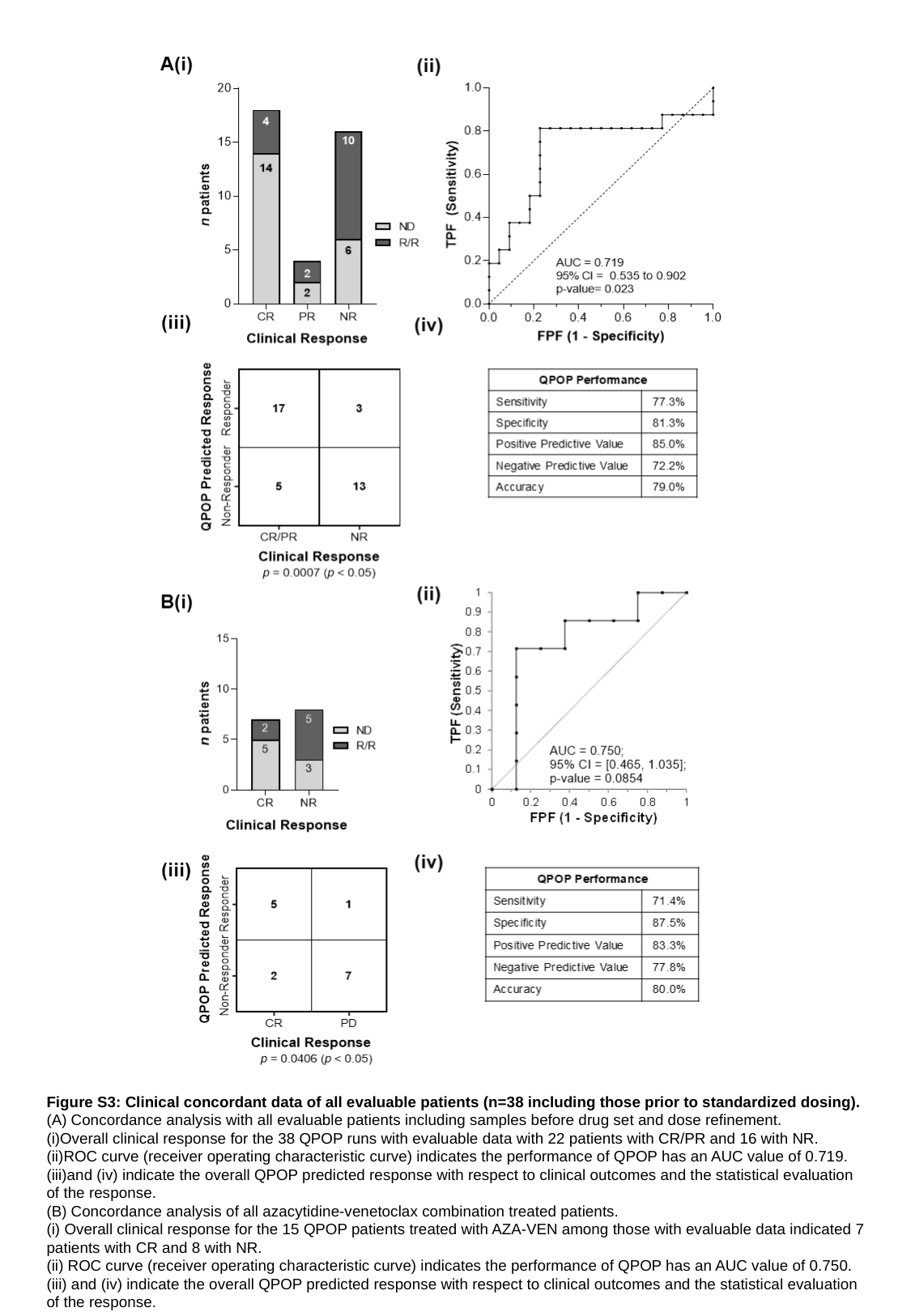

Figure S3: Clinical concordant data of all evaluable patients (n=38 including those prior to standardized dosing).
(A) Concordance analysis with all evaluable patients including samples before drug set and dose refinement.
Overall clinical response for the 38 QPOP runs with evaluable data with 22 patients with CR/PR and 16 with NR.
ROC curve (receiver operating characteristic curve) indicates the performance of QPOP has an AUC value of 0.719.
and (iv) indicate the overall QPOP predicted response with respect to clinical outcomes and the statistical evaluation of the response.
(B) Concordance analysis of all azacytidine-venetoclax combination treated patients.
(i) Overall clinical response for the 15 QPOP patients treated with AZA-VEN among those with evaluable data indicated 7 patients with CR and 8 with NR.
(ii) ROC curve (receiver operating characteristic curve) indicates the performance of QPOP has an AUC value of 0.750. (iii) and (iv) indicate the overall QPOP predicted response with respect to clinical outcomes and the statistical evaluation of the response.

## Slide 9
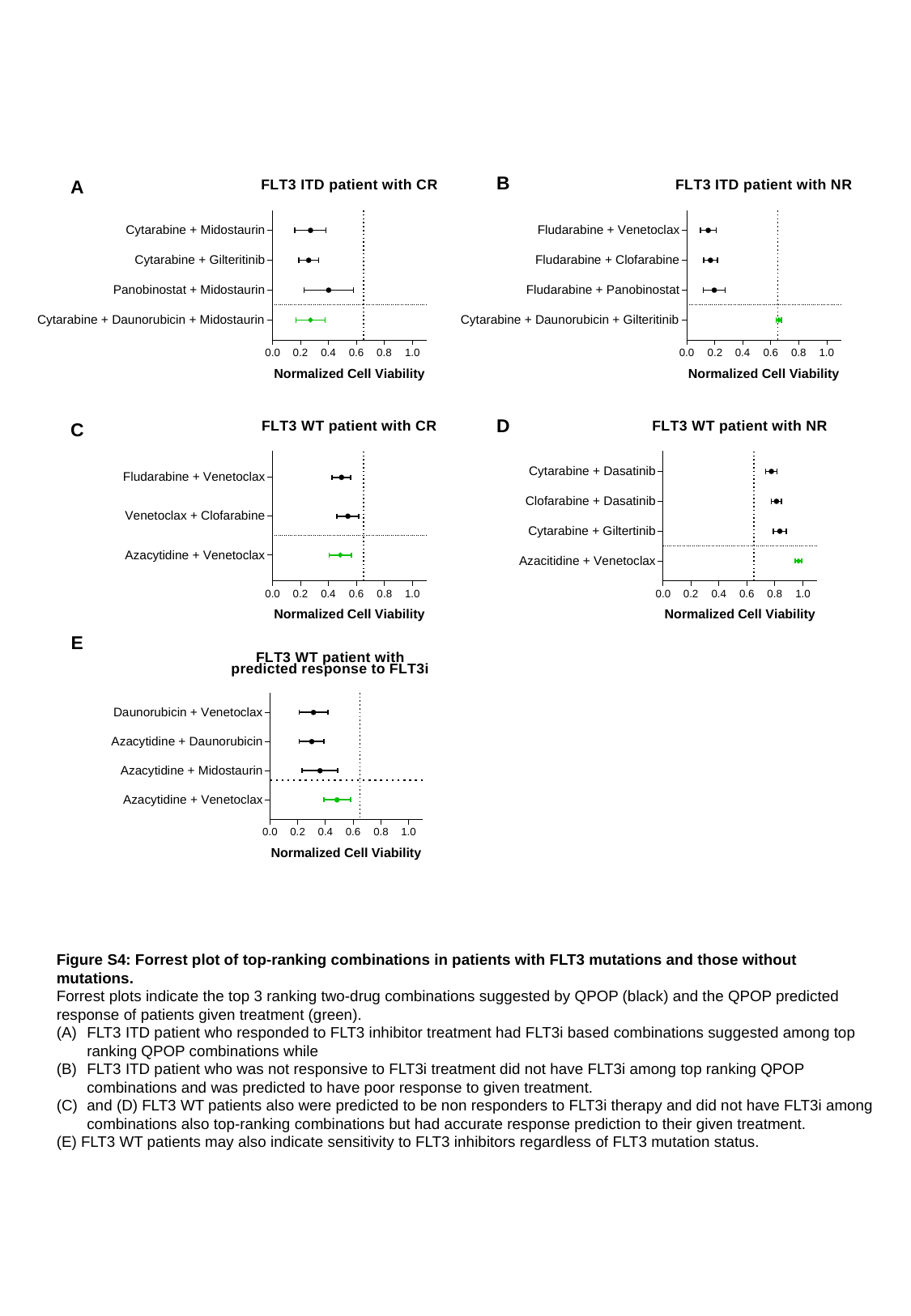

B
A
D
C
E
Figure S4: Forrest plot of top-ranking combinations in patients with FLT3 mutations and those without mutations.
Forrest plots indicate the top 3 ranking two-drug combinations suggested by QPOP (black) and the QPOP predicted response of patients given treatment (green).
FLT3 ITD patient who responded to FLT3 inhibitor treatment had FLT3i based combinations suggested among top ranking QPOP combinations while
FLT3 ITD patient who was not responsive to FLT3i treatment did not have FLT3i among top ranking QPOP combinations and was predicted to have poor response to given treatment.
and (D) FLT3 WT patients also were predicted to be non responders to FLT3i therapy and did not have FLT3i among combinations also top-ranking combinations but had accurate response prediction to their given treatment.
(E) FLT3 WT patients may also indicate sensitivity to FLT3 inhibitors regardless of FLT3 mutation status.

## Slide 10
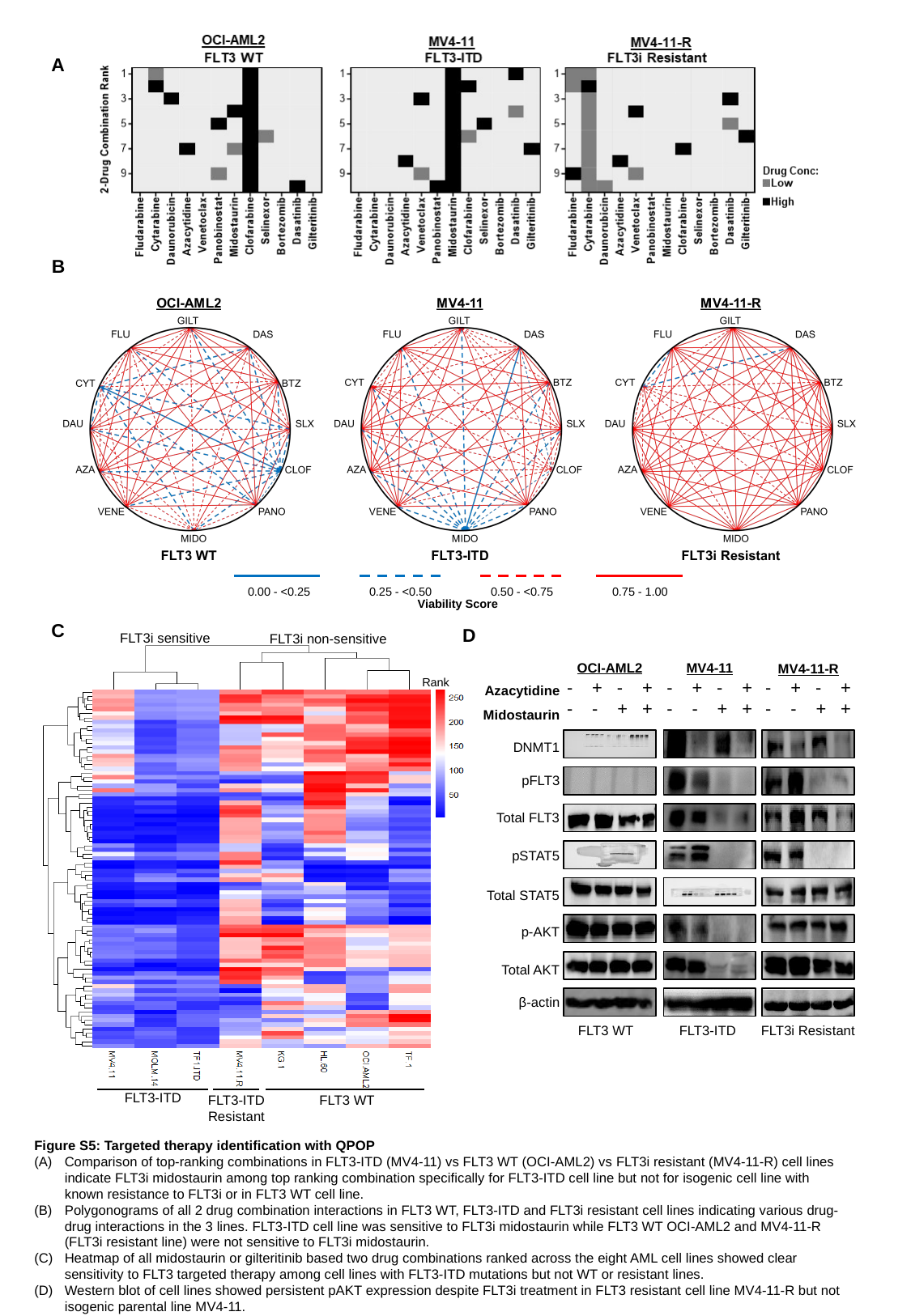

A
B
0.00 - <0.25 	0.25 - <0.50	0.50 - <0.75 	0.75 - 1.00
Viability Score
C
FLT3i sensitive
FLT3i non-sensitive
Rank
FLT3-ITD
FLT3-ITD Resistant
FLT3 WT
D
OCI-AML2
- + - +
- - + +
FLT3 WT
MV4-11
- + - +
- - + +
FLT3-ITD
MV4-11-R
- + - +
- - + +
FLT3i Resistant
Azacytidine
Midostaurin
DNMT1
pFLT3
Total FLT3
pSTAT5
Total STAT5
p-AKT
Total AKT
β-actin
Figure S5: Targeted therapy identification with QPOP
Comparison of top-ranking combinations in FLT3-ITD (MV4-11) vs FLT3 WT (OCI-AML2) vs FLT3i resistant (MV4-11-R) cell lines indicate FLT3i midostaurin among top ranking combination specifically for FLT3-ITD cell line but not for isogenic cell line with known resistance to FLT3i or in FLT3 WT cell line.
Polygonograms of all 2 drug combination interactions in FLT3 WT, FLT3-ITD and FLT3i resistant cell lines indicating various drug-drug interactions in the 3 lines. FLT3-ITD cell line was sensitive to FLT3i midostaurin while FLT3 WT OCI-AML2 and MV4-11-R (FLT3i resistant line) were not sensitive to FLT3i midostaurin.
Heatmap of all midostaurin or gilteritinib based two drug combinations ranked across the eight AML cell lines showed clear sensitivity to FLT3 targeted therapy among cell lines with FLT3-ITD mutations but not WT or resistant lines.
Western blot of cell lines showed persistent pAKT expression despite FLT3i treatment in FLT3 resistant cell line MV4-11-R but not isogenic parental line MV4-11.

## Slide 11
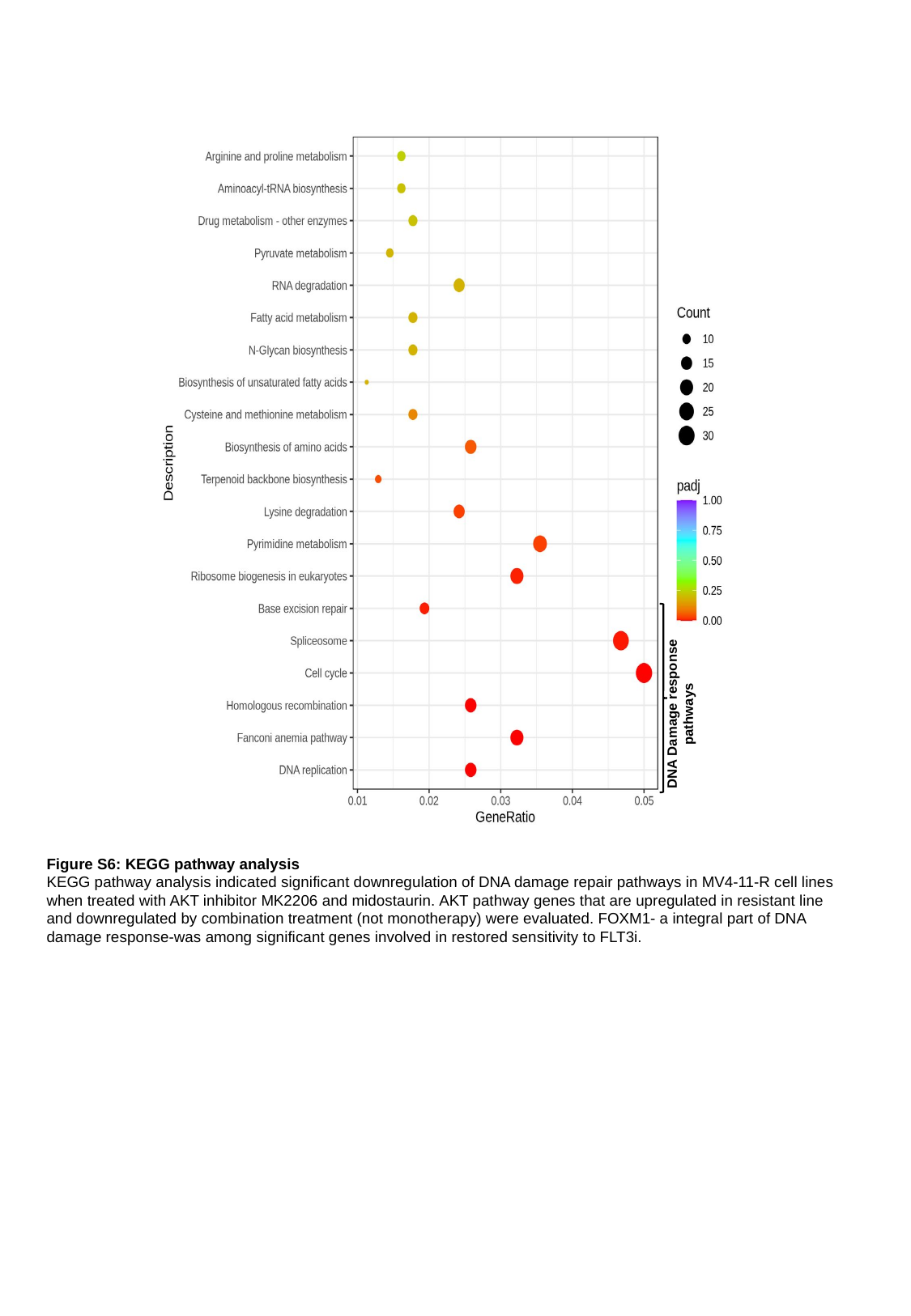

DNA Damage response pathways
Figure S6: KEGG pathway analysis
KEGG pathway analysis indicated significant downregulation of DNA damage repair pathways in MV4-11-R cell lines when treated with AKT inhibitor MK2206 and midostaurin. AKT pathway genes that are upregulated in resistant line and downregulated by combination treatment (not monotherapy) were evaluated. FOXM1- a integral part of DNA damage response-was among significant genes involved in restored sensitivity to FLT3i.

## Slide 12
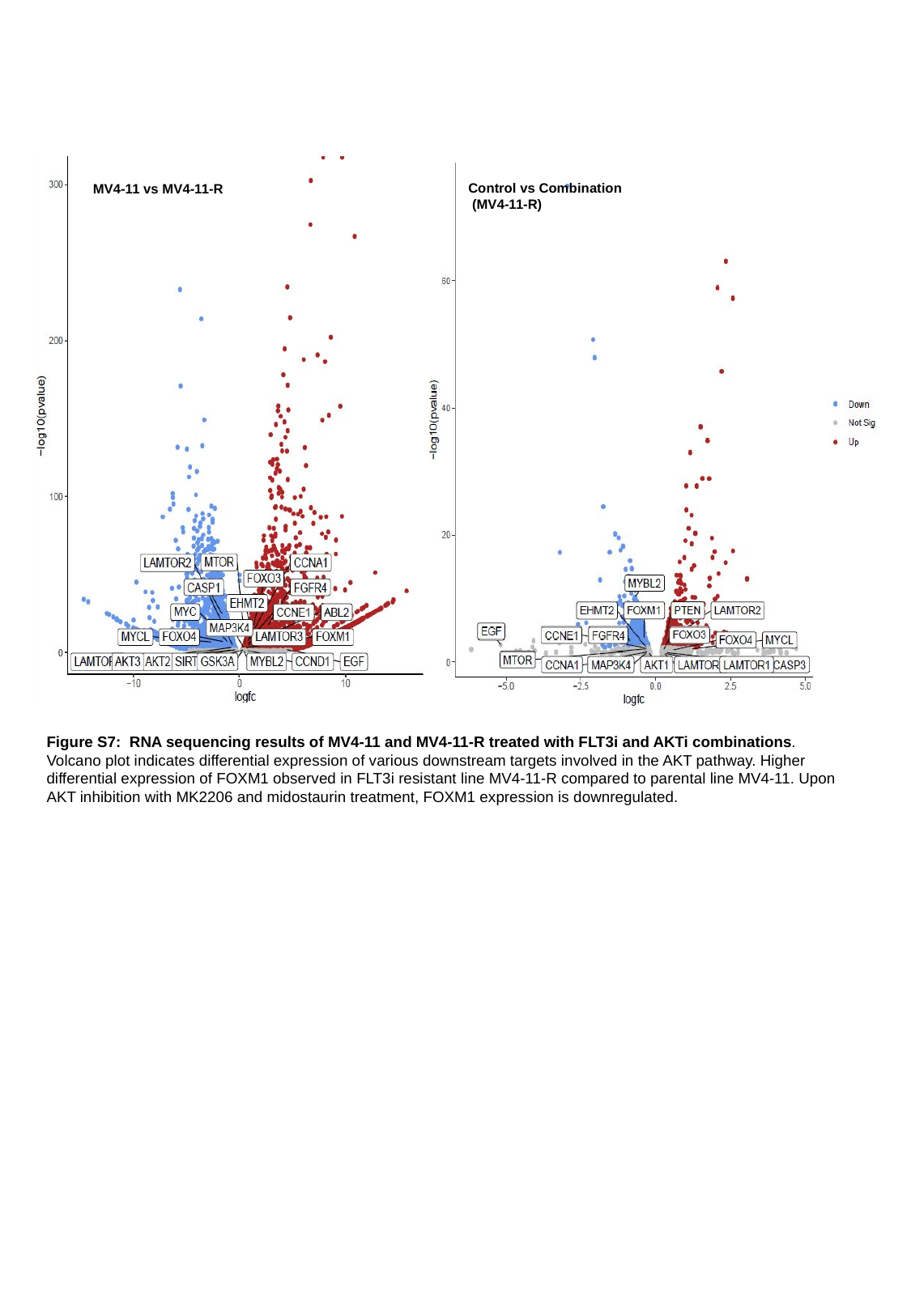

MV4-11 vs MV4-11-R
Control vs Combination
 (MV4-11-R)
Figure S7: RNA sequencing results of MV4-11 and MV4-11-R treated with FLT3i and AKTi combinations.
Volcano plot indicates differential expression of various downstream targets involved in the AKT pathway. Higher differential expression of FOXM1 observed in FLT3i resistant line MV4-11-R compared to parental line MV4-11. Upon AKT inhibition with MK2206 and midostaurin treatment, FOXM1 expression is downregulated.
